# Self-monitoring of Blood Pressure in Patients With Hypertension-Related Multi-morbidity: Systematic Review and Individual Patient Data Meta-analysis

**DOI:** 10.1093/ajh/hpz182

**Published:** 2019-11-15

**Authors:** J P Sheppard, K L Tucker, W J Davison, R Stevens, W Aekplakorn, H B Bosworth, A Bove, K Earle, M Godwin, B B Green, P Hebert, C Heneghan, N Hill, F D R Hobbs, I Kantola, S M Kerry, A Leiva, D J Magid, J Mant, K L Margolis, B McKinstry, M A McLaughlin, K McNamara, S Omboni, O Ogedegbe, G Parati, J Varis, W J Verberk, B J Wakefield, R J McManus

**Affiliations:** 1 Nuffield Department of Primary Care, University of Oxford, Oxford, United Kingdom; 2 Ageing and Stroke Medicine, Norwich Medical School, University of East Anglia, United Kingdom; 3 Department of Community Medicine, Faculty of Medicine, Ramathibodi Hospital, Mahidol University Bangkok, Thailand; 4 Center for Health Services Research in Primary Care, Department of Population Health Sciences, Duke University, Durham, North Carolina, USA; 5 Cardiology, Lewis Katz School of Medicine, Temple University, Philadelphia, Pennsylvania, USA; 6 Thomas Addison Diabetes Unit, St. George’s University Hospitals NHS Foundation Trust, London, United Kingdom; 7 Family Medicine, Memorial University of Newfoundland, St. John’s, Canada; 8 Kaiser Permanente Washington Health Research Institute, Seattle, Washington, USA; 9 Department of Health Services, University of Washington School of Public Health, Seattle, Washington, USA; 10 Division of Medicine, Turku University Hospital and University of Turku, Turku, Finland; 11 Centre for Primary Care and Public Health, Queen Mary University of London, London, United Kingdom; 12 Primary Care Research Unit of Mallorca, Baleares Health Services-IbSalut, Mallorca, Spain; 13 Colorado School of Public Health, University of Colorado, Denver, Colorado, USA; 14 Primary Care Unit, Department of Public Health and Primary Care, University of Cambridge, Cambridge, United Kingdom; 15 HealthPartners Institute, Minneapolis, Minnesota, USA; 16 Usher Institute of Population Health Sciences and Informatics, University of Edinburgh, Edinburgh, United Kingdom; 17 Icahn School of Medicine at Mount Sinai New York, New York, New York, USA; 18 Centre for Medicine Use and Safety, Faculty of Pharmacy and Pharmaceutical Sciences, Monash University, Parkville, Australia; 19 School of Medicine, Deakin University, Geelong, Australia; 20 Clinical Research Unit, Italian Institute of Telemedicine, Varese, Italy; 21 Scientific Research Department of Cardiology, Science and Technology Park for Biomedicine, Sechenov First Moscow State Medical University, Moscow, Russian Federation; 22 Center for Healthful Behavior Change, Division of Health and Behavior, Department of Population Health, Langone School of Medicine, New York University, New York, USA; 23 Istituto Auxologico Italiano, IRCCS, Department of Cardiovascular, Neural and Metabolic Sciences, San Luca Hospital, Milan, Italy; 24 Department of Medicine and Surgery, University of Milano-Bicocca, Milan, Italy; 25 Cardiovascular Research Institute Maastricht and Departments of Internal Medicine, Maastricht University, Maastricht, The Netherlands; 26 Department of Veterans (VA) Health Services Research and Development Centre for Comprehensive Access and Delivery Research and Evaluation (CADRE), VA Medical Centre, Iowa City, USA

**Keywords:** blood pressure, coronary heart disease, diabetes, hypertension, obesity, randomized controlled trial, stroke

## Abstract

**BACKGROUND:**

Studies have shown that self-monitoring of blood pressure (BP) is effective when combined with co-interventions, but its efficacy varies in the presence of some co-morbidities. This study examined whether self-monitoring can reduce clinic BP in patients with hypertension-related co-morbidity.

**METHODS:**

A systematic review was conducted of articles published in Medline, Embase, and the Cochrane Library up to January 2018. Randomized controlled trials of self-monitoring of BP were selected and individual patient data (IPD) were requested. Contributing studies were prospectively categorized by whether they examined a low/high-intensity co-intervention. Change in BP and likelihood of uncontrolled BP at 12 months were examined according to number and type of hypertension-related co-morbidity in a one-stage IPD meta-analysis.

**RESULTS:**

A total of 22 trials were eligible, 16 of which were able to provide IPD for the primary outcome, including 6,522 (89%) participants with follow-up data. Self-monitoring was associated with reduced clinic systolic BP compared to usual care at 12-month follow-up, regardless of the number of hypertension-related co-morbidities (−3.12 mm Hg, [95% confidence intervals −4.78, −1.46 mm Hg]; *P* value for interaction with number of morbidities = 0.260). Intense interventions were more effective than low-intensity interventions in patients with obesity (*P* < 0.001 for all outcomes), and possibly stroke (*P* < 0.004 for BP control outcome only), but this effect was not observed in patients with coronary heart disease, diabetes, or chronic kidney disease.

**CONCLUSIONS:**

Self-monitoring lowers BP regardless of the number of hypertension-related co-morbidities, but may only be effective in conditions such obesity or stroke when combined with high-intensity co-interventions.

Hypertension is the most common individual condition in patients with multi-morbidity.^1^ Multi-morbidity is defined at having two or more concomitant medical conditions and affects between 10% and 50% of patients, depending on the population studied.^1–4^ Increasing multi-morbidity is associated with reduced quality of life.^5,6^ Due to the complexities of studying individuals with multiple conditions, few studies have examined interventions specifically designed to improve outcomes in patients with multi-morbidity.^7^

Optimal management of blood pressure (BP) represents the most effective way to prevent stroke and cardiovascular disease.^8^ Self-monitoring and self-management of BP are effective in reducing BP in patients with hypertension.^9^ However, in patients with multi-morbidity, it is possible that such interventions may be less effective due to clinical inertia on the part of the treating physician^10,11^ or patient concerns about self-monitoring in the presence of certain co-morbidities.^12^ Existing studies have failed to show that self-management can result in improvement in risk factor management in patients with multi-morbidity^13,14^ and individual trials usually contain too few individuals with multi-morbidity to examine outcomes with adequate power, particularly in subgroups.

The BP-SMART collaboration previously carried out an individual patient data (IPD) meta-analysis of trials examining the efficacy of self-monitoring of BP, including data from 25 studies and 8,931 patients.^15,16^ This analysis showed reductions in BP with self-monitoring which increased with the intensity of co-intervention. However, pre-specified subgroup analyses suggested that in some individuals with hypertension-related co-morbidity, such as stroke or myocardial infarction, this effect may be reduced.^15^ To better understand the effect of self-monitoring on clinic BP in a population with multi-morbidity, we systematically reviewed the literature for new trials and undertook IPD meta-analyses by number and type of hypertension-related co-morbidities. In contrast to our previous work, the present study aimed to account for the modifying effect of intensity of co-intervention in analysis of subgroups, which has been shown to be important in determining the efficacy of self-monitoring.^15^ Hypertension was considered as the illness, along with co-morbidities commonly associated with hypertension (coronary heart disease [CHD], stroke [including transient ischemic attack], diabetes, chronic kidney disease [CKD; defined as stage 3a or above], and obesity [body mass index of ≥30 kg/m^2^]).

## METHODS

### Study design

This work extends a previous systematic review and IPD analysis of self-monitoring of BP in hypertensive patients.^15,16^ Searches of the literature were undertaken to identify new trials published since the previous review providing data on the efficacy of self-monitoring of BP which could be combined with data from the original BP-SMART collaboration.^15,16^ Where available, these data were combined and analyzed in a one-stage IPD meta-analysis.

### Data sources and searches

A previously published search conducted in Medline, Embase, and the Cochrane Library^15^ was updated to identify trials examining the efficacy of self-monitoring of BP in hypertensive patients, published up to January 2018 (Supplementary Figure S1).

### Study selection

At least two reviewers (KT, RM, and WD) independently assessed the articles for eligibility and inclusion, disagreements were resolved by discussion. All published and unpublished controlled trials included in the analysis were required to fulfill the following criteria:

- **Population**: patients with hypertension, not being managed as an inpatient.
- **Intervention**: self-measurement of BP without medical professional input plus or minus other co-interventions.
- **Comparator**: no organized self-measurement of BP, although there may be some *ad hoc* measurement which would be difficult to prevent or assess.
- **Outcome**: systolic and/or diastolic BP measured in clinic, or by daytime ambulatory measurement.
- **Study design**: randomized trial of at least 100 participants followed up for at least 24 weeks (to ensure a minimum level of study quality and robustness of effect estimates).
- **Publication Date**: since 2000 (because changes in the technology used for self-monitoring make comparisons prior to this date less relevant).

All articles were managed and screened using the Covidence application (Veritas Health Innovation Ltd, Melbourne, Australia).

### Data extraction and quality assessment

Corresponding authors whose trials met the inclusion criteria were approached for provision of IPD including demographic details, antihypertensive medications, lifestyle factors, and BP end points (clinic and/or ambulatory). All patients had hypertension, and data regarding other morbidities were also sought. This analysis focused on morbidities commonly associated with hypertension (CHD, stroke, diabetes, CKD, and obesity), since recording of such data varied widely across trials and only these conditions commonly were captured frequently enough to enable data to pooled in this analysis. Where data on even these conditions were missing, the morbidity was assumed not to be present in the population from that particular study (morbidities recorded by each study are listed in Supplementary Table S1). Study-level data were extracted from published articles and checked by the original authors. In particular, any co-interventions were carefully documented and prospectively (prior to conducting the analysis) allocated to one of four levels of interventional support based on a previous classification (Table 1).^15–17^ Due to limited sample sizes for the subgroup analyses planned in the present study, these classifications were condensed into two levels (low vs. high intensity) (Table 1). Study quality was assessed in terms of potential bias from randomization, blinding, outcome assessment, and method of analysis using an adaptation of the Cochrane risk of bias tool.^15^ Original data were kept on a secure server and re-coded to a consistent format across trials, where appropriate.

**Table 1.** Definitions of high- and low-level intensity co-interventions

	Level	Name	Description
Low-intensity intervention	Level 1	Self-monitoring with minimal additional contact	Self-monitoring with one off educational materials and initial instructions from a nurse.
	Level 2	Self-monitoring with automated feedback or support	Web based or telephonic tools provide feedback or support. But no regular 1:1 contact.
High-intensity intervention	Level 3	Self-monitoring with an active intervention	Web based or telephonic tools provide feedback or support and education offered in regular classes. No regular 1:1 contact.
	Level 4	Self-monitoring with significant tailored support	Individually tailored support from study personnel, pharmacist or a clinician. Could include checking BP/medication or education/lifestyle counseling.

This was based on previous work by Uhlig *et al.*^17^ and Tucker *et al*.^15^

### Outcome measures

The primary outcome was change in clinic BP (systolic and diastolic) between baseline and 12-month follow-up, by number of morbidities. Secondary analyses examined the likelihood of uncontrolled BP (as defined by the original study; determined by the study population and setting [see Supplementary Table S1 for BP targets]) at 12 months by number of co-morbidities. All outcomes were also assessed at 6-month follow-up. Further analyses explored subgroups by type of co-morbidity (in addition to hypertension: CHD, stroke, diabetes, CKD, and obesity) and intensity of intervention (high vs. low intensity).

### Data synthesis and analysis

Descriptive statistics were used to summarize the baseline characteristics of included patients by type of hypertension-related co-morbidity. The overall impact of self-monitoring on BP was assessed in a two-stage IPD meta-analysis. For outcomes by co-morbidity, a one-stage IPD meta-analysis was conducted with both random intercept and random coefficients to account for study-level effects and heterogeneity in treatment effects across studies. Linear regression was used for continuous outcomes (change in systolic and diastolic BP) and logistic regression for binary outcomes (odds of uncontrolled BP at follow-up). All analyses were conducted by intention-to-treat and each model was adjusted for age, sex, baseline clinic BP and level of intervention.

Subgroup analyses were used to examine the effect of self-monitoring on change in BP and likelihood of uncontrolled BP in patients with CHD, stroke, diabetes, CKD, and obesity. In each model, the interaction between self-monitoring and intensity of co-intervention was explored (high vs. low intensity; defined in Table 1). Sensitivity analyses were conducted to examine the impact of missing studies by including published aggregate data from those trials which were not able to provide IPD for this review. Funnel plots and Egger’s test^18^ were used to assess the potential for publication bias.

All analyses were conducted using STATA version 14.1 (Special Edition, StataCorp, College Station, TX). Data are presented as proportions of the total study population, means with standard deviation with 95% confidence intervals unless otherwise stated.

### Role of the funding source

The funders played no role in the design or execution of the study and no role in the preparation of this manuscript. The views expressed are those of the author(s) and not necessarily those of the NHS, the NIHR, or the Department of Health and Social Care.

## RESULTS

The previous literature review identified 36 studies for which data from 25 randomized controlled trials were obtained.^15^ The updated search conducted for this analysis returned 1,377 new studies (Supplementary Figure S2) and after title and abstract screening, 32 full text articles were assessed. In total, three new trials were identified as eligible for inclusion in the BP-SMART database. Of these, one provided IPD and the remaining two studies were unable to provide data or did not respond (Supplementary Figure S2).

The total dataset included 26 studies published between 2005 and 2016 including data from 10,713 participants (Supplementary Table S1). Data for the primary outcome (change in clinic BP at 12 months) were available in 16 studies and 7,360 participants, of which 6,522 (88.6%) had complete follow-up data and were included in the final analysis.^9,19–42^ On average, self-monitoring reduced clinic BP by 3.11/1.49 mm Hg (systolic/diastolic), although there was significant heterogeneity across studies (*I*^2^ = 59.6–75.4%, *P* <0.001; Supplementary Figures S3 and S4). Inclusion of aggregate data from studies which were not able to provide IPD did not affect the overall results (Supplementary Figure S5). There was no evidence of publication bias among studies included in this review (Egger’s test^18^ = 0.07, *P*=0.977; Supplementary Figure S6).

Patients had between 1 and 6 morbidities (median 2, interquartile range 1.2) including hypertension, which was present in all participants (Supplementary Table S2). The characteristics of patients with different hypertension-related co-morbidities were broadly similar, although patients with a history of CHD and stroke were older and those with diabetes were more commonly male, with a higher proportion of smokers and were prescribed more BP lowering medications at baseline (Supplementary Table S2).

### Effect of self-monitoring by number of hypertension-related co-morbidities

**Figure 1.**
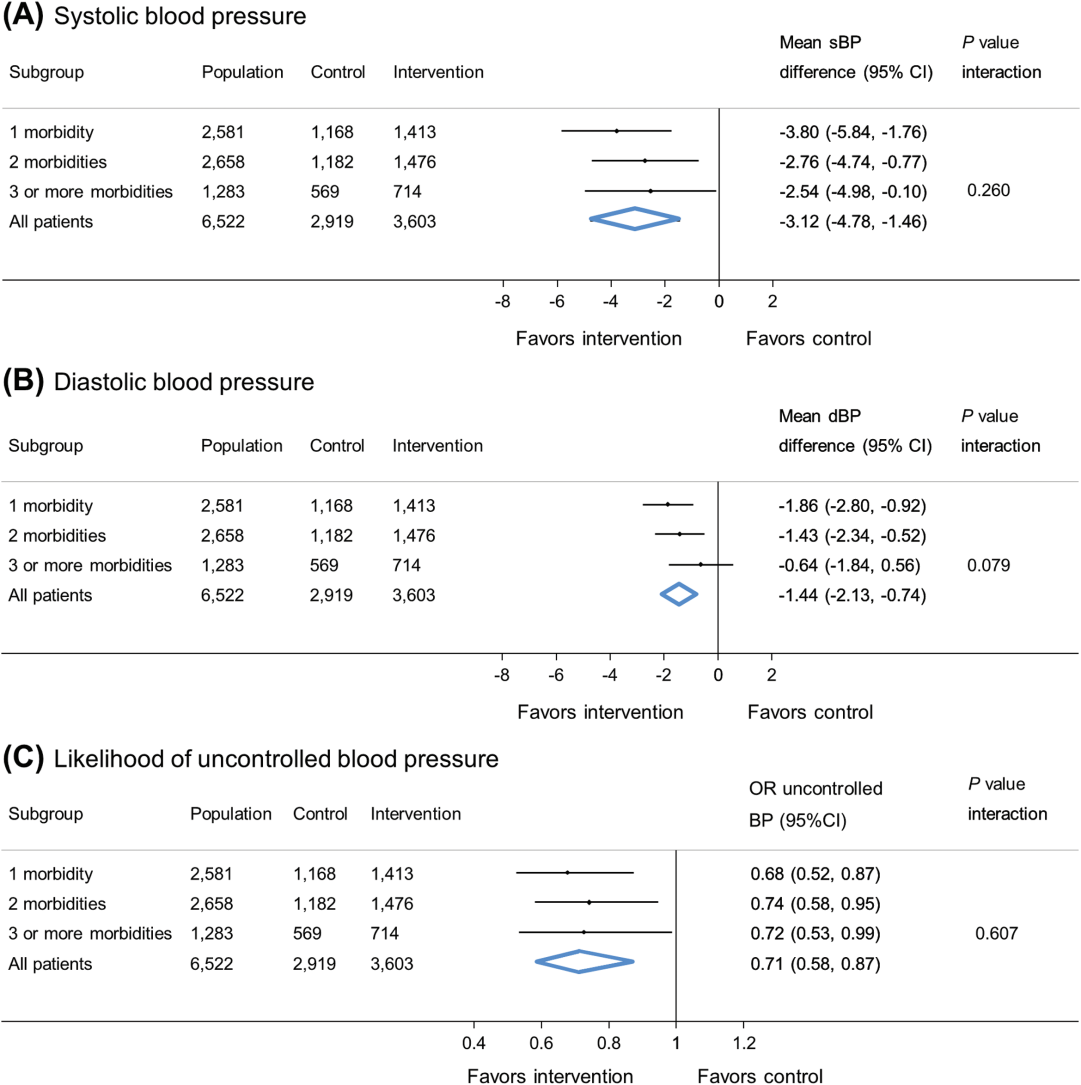
Effect of self-monitoring on clinic blood pressure at 12-month follow-up by number of hypertension-related co-morbidities (16 studies). Blood pressure difference given in mm Hg. Analyses adjusted for age, sex, baseline blood pressure, and level of intervention, with study-level random effects for intervention and usual care. Abbreviations: SBP, systolic blood pressure; DBP, diastolic blood pressure; CI, confidence intervals; OR, odds ratio. Uncontrolled blood pressure defined by thresholds specified in each contributing study (see Supplementary Table S2 for details).

In patients with hypertension but no other hypertension-related co-morbidities, self-monitoring was associated with a 3.80 mm Hg reduction (95% confidence intervals [CI] 5.84, 1.76 mm Hg) in clinic systolic BP and 1.86 mm Hg reduction (95% CI 2.80, 0.92 mm Hg) in clinic diastolic BP at 12-month follow-up (Figure 1). The was no difference in the effectiveness of self-monitoring by increasing numbers of co-morbidities (systolic blood pressure *P* for interaction = 0.260; diastolic blood pressure *P* for interaction = 0.079). Self-monitoring of BP was associated with reduced odds of having uncontrolled clinic BP at 12-month follow-up (OR [odds ratio] 0.68, 95% CI 0.52, 0.87), and this was similar in patients with increasing numbers of co-morbidities (*P* for interaction = 0.607). Similar findings were observed at 6-month follow-up (Supplementary Figure S7).

### Effect of self-monitoring by intervention intensity within specific morbidities

**Figure 2.**
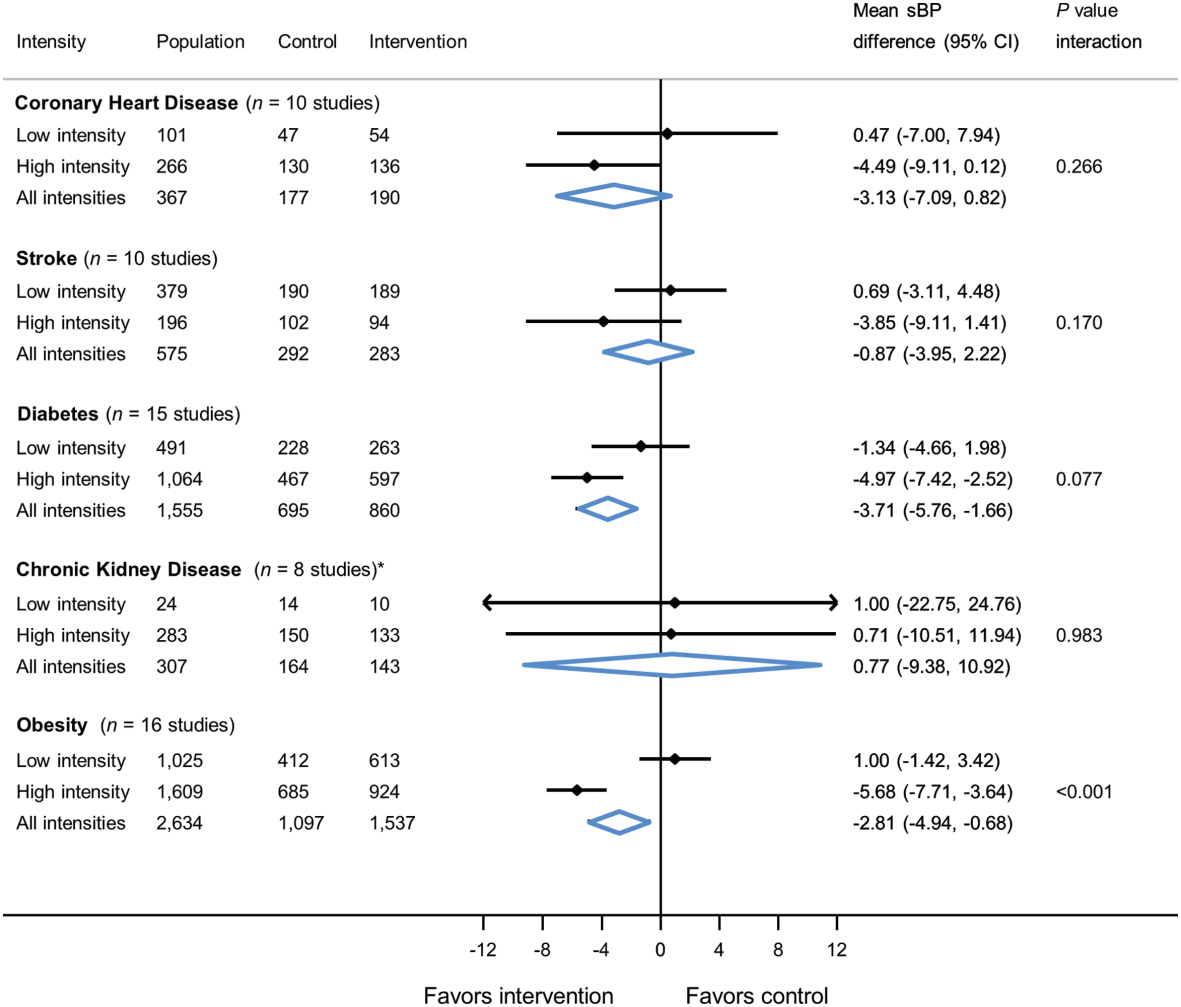
Effect of self-monitoring on clinic systolic blood pressure at 12-month follow-up by intervention intensity within specific morbidities. *Two studies only provided one patient each to the model. Blood pressure difference given in mm Hg. Analyses adjusted for age, sex, and baseline blood pressure with study-level random effects for intervention and usual care. Abbreviations: sBP, systolic blood pressure; CI, confidence intervals; CHD, coronary heart disease; CKD, chronic kidney disease.

Self-monitoring was associated with lower clinic systolic BP in patients with diabetes (−3.71 mm Hg, 95% CI −5.76, −1.66 mm Hg) and obesity (−2.81 mm Hg, 95% CI −4.94, −0.68 mm Hg), but not patients with CHD, stroke, or CKD (Figure 2). There was a significant interaction between the effect of self-monitoring and intervention intensity in patients with obesity (*P* value for interaction = <0.001) (Figure 2). Similar findings were observed for diastolic BP (Supplementary Figure S8) and at 6-month follow-up (Supplementary Figures S9 and S10).

**Figure 3.**
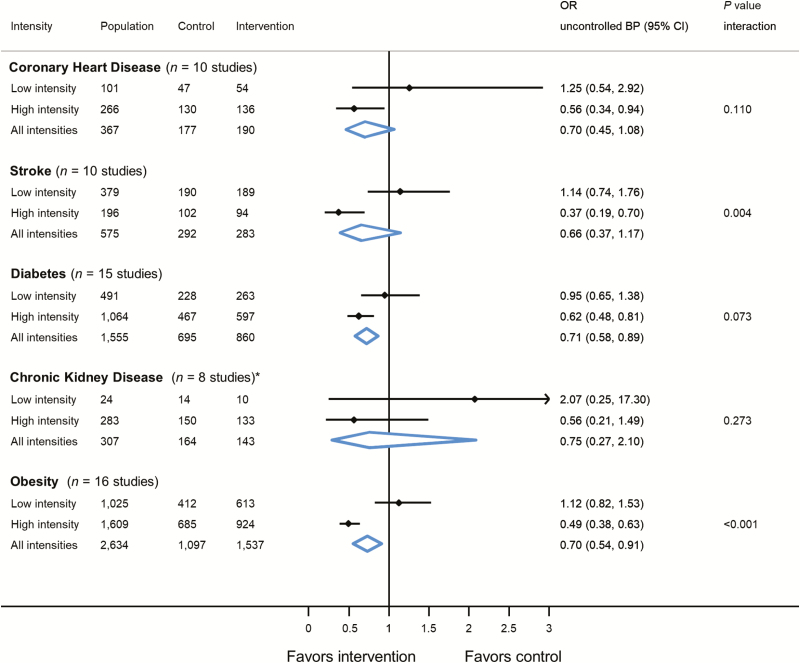
Effect of self-monitoring on likelihood of uncontrolled clinic blood pressure at 12-month follow-up by intervention intensity within specific morbidities. *Two studies only provided one patient each to the model. Analyses adjusted for age, sex and baseline blood pressure with study-level random effects for intervention and usual care. Abbreviations: OR, odds ratio; CI, confidence intervals; CHD, coronary heart disease; CKD, chronic kidney disease. Uncontrolled blood pressure defined by thresholds specified in each contributing study (see Supplementary Table S2 for details).

For patients with diabetes and obesity, self-monitoring reduced the likelihood of uncontrolled clinic BP at 12-month follow-up (Figure 3). A significant interaction between the effect of self-monitoring and intensity of intervention was observed in patients with stroke (OR 1.14, 95% CI 0.74–1.1.76 [low intensity] vs. OR 0.37, 95% CI 0.19–0.70 [high intensity]; interaction = 0.004) and obesity (OR 1.12, 95% CI 0.82–1.53 [low intensity] vs. OR 0.49, 95% CI 0.38–0.63 [high intensity]; interaction = <0.001). At 6-month follow-up, self-monitoring was associated with a reduced likelihood of uncontrolled clinic BP in patients with diabetes, CKD, and obesity (Supplementary Figure S11). In patients stroke, diabetes, CKD, and obesity, there was a significant interaction between the effect of self-monitoring and intensity of intervention, with those receiving high-intensity interventions being less likely to have uncontrolled clinic BP at 6-month follow-up.

## DISCUSSION

### Summary of findings

This is the largest IPD meta-analysis to date of self-monitoring in hypertension including IPD from 6,522 patients and 16 trials of self-monitoring of BP in hypertension.^9,19–42^ Self-monitoring was found to be effective at lowering BP,^15,17,43^ and this effect was observed regardless of the number of hypertension-related co-morbidities present. This study confirms that self-monitoring is effective in patients with obesity.^15^ In contrast to previous studies, there was some limited evidence that patients with stroke may benefit from self-monitoring when it is combined with a high-intensity co-intervention. Such co-interventions might include self-management, pharmacist support, tailored education, and lifestyle counseling. Self-monitoring of BP can therefore be recommended as part of a multifaceted approach to managing hypertensive patients with hypertension-related co-morbidity.

### Strengths and weaknesses

This is the largest, and to our knowledge only, IPD meta-analysis of trials examining the efficacy of self-monitoring of BP in hypertensive patients with a hypertension-related co-morbidity. Having access to IPD provided a unique opportunity to study the effect of self-monitoring within specific morbidities, something which is not possible in standard meta-analyses.^17^ As is common in this type of review, it was not possible to obtain data from all eligible studies, due to inability to make contact with authors, or data no longer being held in a format that could be transferred across institutions and analyzed. Despite this, complete follow-up data were available from 6,522 participants in 16 studies that provided data on the primary outcome (at 12-month follow-up). Our sensitivity analyses suggest that missing studies would have had little impact on the overall association between self-monitoring and BP. Because our analyses examined the number and type of hypertension-related co-morbidity, it was not possible to combine IPD with aggregate data from unavailable trials (where patients have varying morbidities) to examine the impact of these missing data on our hypertension-related co-morbidity subgroups.

The focus of this analysis was on the extent to which hypertension-related co-morbidity modifies the effect of self-monitoring on BP. Co-morbidities were characterized in terms of 6 conditions related to hypertension (hypertension, diabetes, CKD, CHD, stroke, and obesity) for which sufficient data were available. However, some included studies did not collect information about these conditions (see Supplementary Table S1 for details), which may have led to an under representation of the prevalence of each condition in the study cohort. In addition, there are many other co-morbidities that can be used to define multi-morbidity^1^ and may have been present in some patients but were not captured as part of the original studies contributing data to these analyses.

For the present study, we developed a one-stage analytical model with study-level random effects for each intervention and control group. In contrast, our previous analysis included a single study-level covariate which gave less weight to the individual study effects and potentially underestimated the between study variance. This change in analytical approach had little effect in most of our analyses, except that which examined patients with CKD. In that analysis one study (contributing 15 patients)^42^ suggested that self-monitoring increases systolic BP by 41.2 mm Hg, compared to the remaining 7 studies (contributing 292 patients) which showed a 5.1 mm Hg reduction at 12-month follow-up. Since the present analysis gives more weight to individual studies, our combined findings were drawn toward the null whereas in our previous paper they were not.^15^ Such subgroup analyses, with very small sample sizes and imprecise point estimates should be interpreted with caution. Indeed, differences between results at 6-month and 12-month follow-up could be explained by the larger number of studies and participants available for assessment of outcomes at 6-month follow-up.

The nature of interventions categorized as high and low intensity were quite heterogeneous and significantly more patients and trials would be required to identify exactly which type of co-interventions is most effective in which condition. Included studies had rates of follow-up which varied between 58% and 99% with most studies following-up around 90% of participants. Our previous analysis using this dataset suggested the impact of differential follow-up in individual studies was negligible.^15^

Studies included in this review used various different measurement protocols for both clinic and home BP readings (e.g., number of readings, days, period of rest prior to measurement, etc.). Where individual BP readings were available from each included study, the definition of clinic and home BP was standardized (clinic BP = mean of the second and third readings; home BP = mean of 6 days of readings, after discarding the first day’s readings). However, for the majority of studies this standardization was not possible. Whilst this may have affected the absolute values for BP reported in each trial, we do not think this would have affected the overall findings, since each randomized group were subjected to the same measurement procedures within each study. Our analyses also took into account random treatment effects across studies, which could include those brought about by varying measurement protocols between studies.

### Comparison with previous literature

The efficacy of self-monitoring in patients with multi-morbidity has been debated, with some studies suggesting it may be beneficial,^13,14^ and others questioning its effectiveness in specific morbidities.^29^ This study confirms the beneficial effects of self-monitoring of BP in hypertension-related co-morbidity and patients with specific conditions such as obesity and demonstrates possible effects in stroke, highlighting the importance of intensity of co-intervention for certain conditions. This study is novel in comparison with our previous review^15^ due to the inclusion of additional data, better characterization of multi-morbidity within studies and updated analysis taking into account the intensity of co-intervention within subgroups.

Previous reviews have attempted to define the effects of self-monitoring as part of a wider self-management intervention, in patients with diabetes and CKD,^44^ and those with previous stroke.^45^ The present analysis included nearly four times as many patients with diabetes and/or CKD but was still underpowered to show whether self-monitoring is effective at reducing BP when combined with co-interventions such as self-management or 1:1 counseling in patients with specific morbidities. Where more data were available at 6-month follow-up, and examining the likelihood of uncontrolled BP rather than change in BP, there was some evidence to suggest that self-monitoring is effective in patients with stroke, diabetes, CKD, and obesity, in combination with high-intensity co-interventions. This latter finding was also seen in patients with stroke and obesity at 12-month follow-up.

### Implications for practice

Many previous studies have considered the impact of self-monitoring in hypertension,^15^ or patients with specific morbidities.^23,29,40^ However in practice, patients present with multiple morbidities and so it is important to consider the efficacy of self-monitoring in the context of multi-morbidity. The findings of this review suggest that self-monitoring can be recommended as part of a multifaceted approach to managing hypertensive patients with hypertension-related co-morbidity. There was some variation in the effectiveness of self-monitoring within specific morbidities, and this can only be partly explained by the use of high- vs. low-intensity interventions. However, the present findings suggest that where individuals have a history of obesity and possibly stroke, self-monitoring is likely to be effective when combined with intensive co-interventions such as self-management, pharmacist support, tailored education or lifestyle advice. Understanding the relative cost effectiveness of the different co-interventions is likely to be important when deciding which should be encouraged in routine practice. The present analysis suggests that targeting individuals with hypertension-related co-morbidity is appropriate and this may make the financial case for costlier interventions stronger, since patients with such co-morbidities are at greater risk of cardiovascular disease.^1^ Further work should use these IPD to quantify the impact of self-monitoring on outcomes other than BP, as others have attempted using aggregated data in previous reviews.^45^

Self-monitoring of BP leads to clinically significant BP reductions in patients with hypertension-related co-morbidity and can recommended as part of a wider management plan in routine clinical practice. Some limited evidence suggests that patients with stroke and/or obesity should be targeted for self-monitoring interventions that are combined with systematic medication titration, pharmacist support, education, or lifestyle to maximize the likelihood of BP control at follow-up.

## DISCLOSURE

This research was funded by the National Institute for Health Research School for Primary Care Research (NIHR SPCR number 267) and via an National Institute for Health Research Professorship (NIHR-RP-02-12-015). JS holds a Wellcome Trust/Royal Society Sir Henry Dale Fellowship (ref 211182/Z/18/Z). RM, KT and JS have, or previously received funding from the National Institute for Health Research (NIHR) Collaboration for Leadership in Applied Health Research and Care Oxford at Oxford Health NHS Foundation Trust. JM and RMcM are NIHR Senior Investigators. HB received funds from NHLBI (R01 HL070713). KM received funds from National Heart, Lung, and Blood Institute (NHLBI) (R01 HL090965). FDRH acknowledges part support from the NIHR School for Primary Care Research (SPCR), the NIHR Collaboration for Leadership in Applied Research in Health and Care (CLARHC) Oxford, and the NIHR Biomedical Research Centre (BRC), Oxford. RM has received research funding in terms of BP monitors from Omron. RM leads a programme of research around self-monitoring/management in stroke with BMc, funded by the Stroke Association and British Heart Foundation. BMc is the clinical lead for the Scottish Government’s National programme for scaling up tele-monitoring in Scotland. HB receives research funds from Otsuka pharmaceuticals, Novo Nordisk, and Sanofi, but none of these studies are related to the current study. NH is now an employee of Bristol-Myers Squibb. The authors declare no other conflicts of interest. The views expressed are those of the authors and not necessarily those of the NIHR or the Department of Health and Social Care.

## Supplementary Material

hpz182_suppl_Supplementary_Appendix

## References

[1] Barnett K, Mercer SW, Norbury M, Watt G, Wyke S, Guthrie B Epidemiology of multimorbidity and implications for health care, research, and medical education: a cross-sectional study. Lancet 2012; 380:37–43. 22579043 10.1016/S0140-6736(12)60240-2

[2] Wang HH, Wang JJ, Wong SY, Wong MC, Li FJ, Wang PX, Zhou ZH, Zhu CY, Griffiths SM, Mercer SW Epidemiology of multimorbidity in China and implications for the healthcare system: cross-sectional survey among 162,464 community household residents in southern China. BMC Med 2014; 12:188. 25338506 10.1186/s12916-014-0188-0 PMC4212117

[3] Ornstein SM, Nietert PJ, Jenkins RG, Litvin CB The prevalence of chronic diseases and multimorbidity in primary care practice: a PPRNet report. J Am Board Fam Med 2013; 26:518–524. 24004703 10.3122/jabfm.2013.05.130012

[4] Violán C, Foguet-Boreu Q, Roso-Llorach A, Rodriguez-Blanco T, Pons-Vigués M, Pujol-Ribera E, Muñoz-Pérez MÁ, Valderas JM Burden of multimorbidity, socioeconomic status and use of health services across stages of life in urban areas: a cross-sectional study. BMC Public Health 2014; 14:530. 24885174 10.1186/1471-2458-14-530 PMC4060853

[5] Fortin M, Lapointe L, Hudon C, Vanasse A, Ntetu AL, Maltais D Multimorbidity and quality of life in primary care: a systematic review. Health Qual Life Outcomes 2004; 2:51. 15380021 10.1186/1477-7525-2-51 PMC526383

[6] Wang L, Palmer AJ, Cocker F, Sanderson K Multimorbidity and health-related quality of life (HRQoL) in a nationally representative population sample: implications of count versus cluster method for defining multimorbidity on HRQoL. Health Qual Life Outcomes 2017; 15:7. 28069026 10.1186/s12955-016-0580-x PMC5223532

[7] Smith SM, Soubhi H, Fortin M, Hudon C, O’Dowd T Interventions for improving outcomes in patients with multimorbidity in primary care and community settings. Cochrane Database Syst Rev 2012;4:CD006560. 10.1002/14651858.CD006560.pub2 22513941

[8] Lewington S, Clarke R, Qizilbash N, Peto R, Collins R; Prospective Studies Collaboration Age-specific relevance of usual blood pressure to vascular mortality: a meta-analysis of individual data for one million adults in 61 prospective studies. Lancet 2002; 360:1903–1913. 12493255 10.1016/s0140-6736(02)11911-8

[9] McManus RJ, Mant J, Haque MS, Bray EP, Bryan S, Greenfield SM, Jones MI, Jowett S, Little P, Penaloza C, Schwartz C, Shackleford H, Shovelton C, Varghese J, Williams B, Hobbs FD, Gooding T, Morrey I, Fisher C, Buckley D Effect of self-monitoring and medication self-titration on systolic blood pressure in hypertensive patients at high risk of cardiovascular disease: the TASMIN-SR randomized clinical trial. JAMA 2014; 312:799–808. 25157723 10.1001/jama.2014.10057

[10] Moise N, Davidson KW, Chaplin W, Shea S, Kronish I Depression and clinical inertia in patients with uncontrolled hypertension. JAMA Intern Med 2014; 174:818–819. 24615061 10.1001/jamainternmed.2014.115 PMC4013232

[11] Khunti K, Nikolajsen A, Thorsted BL, Andersen M, Davies MJ, Paul SK Clinical inertia with regard to intensifying therapy in people with type 2 diabetes treated with basal insulin. Diabetes Obes Metab 2016; 18:401–409. 26743666 10.1111/dom.12626 PMC5067688

[12] Kenning C, Coventry PA, Gibbons C, Bee P, Fisher L, Bower P Does patient experience of multimorbidity predict self-management and health outcomes in a prospective study in primary care? Fam Pract 2015; 32:311–316. 25715962 10.1093/fampra/cmv002 PMC4445135

[13] Katon WJ, Lin EH, Von Korff M, Ciechanowski P, Ludman EJ, Young B, Peterson D, Rutter CM, McGregor M, McCulloch D Collaborative care for patients with depression and chronic illnesses. N Engl J Med 2010; 363:2611–2620. 21190455 10.1056/NEJMoa1003955 PMC3312811

[14] Lorig KR, Sobel DS, Stewart AL, Brown BW Jr, Bandura A, Ritter P, Gonzalez VM, Laurent DD, Holman HR Evidence suggesting that a chronic disease self-management program can improve health status while reducing hospitalization: a randomized trial. Med Care. 1999; 37:5–14. 10413387 10.1097/00005650-199901000-00003

[15] Tucker KL, Sheppard JP, Stevens R, Bosworth HB, Bove A, Bray EP, Earle K, George J, Godwin M, Green BB, Hebert P, Hobbs FDR, Kantola I, Kerry SM, Leiva A, Magid DJ, Mant J, Margolis KL, McKinstry B, McLaughlin MA, Omboni S, Ogedegbe O, Parati G, Qamar N, Tabaei BP, Varis J, Verberk WJ, Wakefield BJ, McManus RJ Self-monitoring of blood pressure in hypertension: a systematic review and individual patient data meta-analysis. PLoS Med 2017; 14:e1002389. 28926573 10.1371/journal.pmed.1002389 PMC5604965

[16] Tucker KL, Sheppard JP, Stevens R, Bosworth HB, Bove A, Bray EP, Godwin M, Green B, Hebert P, Hobbs FD, Kantola I, Kerry S, Magid DJ, Mant J, Margolis KL, McKinstry B, Omboni S, Ogedegbe O, Parati G, Qamar N, Varis J, Verberk W, Wakefield BJ, McManus RJ Individual patient data meta-analysis of self-monitoring of blood pressure (BP-SMART): a protocol. BMJ Open 2015; 5:e008532. 10.1136/bmjopen-2015-008532 PMC4577873 26373404

[17] Uhlig K, Patel K, Ip S, Kitsios GD, Balk EM Self-measured blood pressure monitoring in the management of hypertension: a systematic review and meta-analysis. Ann Intern Med 2013; 159:185–194. 23922064 10.7326/0003-4819-159-3-201308060-00008

[18] Egger M, Davey Smith G, Schneider M, Minder C Bias in meta-analysis detected by a simple, graphical test. BMJ 1997; 315:629–634. 9310563 10.1136/bmj.315.7109.629 PMC2127453

[19] Aekplakorn W, Suriyawongpaisal P, Tansirisithikul R, Sakulpipat T, Charoensuk P Effectiveness of self-monitoring blood pressure in primary care: a randomized controlled trial. J Prim Care Community Health 2016; 7:58–64. 26574566 10.1177/2150131915614069 PMC5932709

[20] Bosworth HB, Olsen MK, Grubber JM, Neary AM, Orr MM, Powers BJ, Adams MB, Svetkey LP, Reed SD, Li Y, Dolor RJ, Oddone EZ Two self-management interventions to improve hypertension control: a randomized trial. Ann Intern Med 2009; 151:687–695. 19920269 10.1059/0003-4819-151-10-200911170-00148 PMC2892337

[21] Bosworth HB, Olsen MK, McCant F, Harrelson M, Gentry P, Rose C, Goldstein MK, Hoffman BB, Powers B, Oddone EZ Hypertension Intervention Nurse Telemedicine Study (HINTS): testing a multifactorial tailored behavioral/educational and a medication management intervention for blood pressure control. Am Heart J 2007; 153:918–924. 17540191 10.1016/j.ahj.2007.03.004

[22] Bove AA, Homko CJ, Santamore WP, Kashem M, Kerper M, Elliott DJ Managing hypertension in urban underserved subjects using telemedicine—a clinical trial. Am Heart J 2013; 165:615–621. 23537980 10.1016/j.ahj.2013.01.004

[23] Earle K In people with poorly controlled hypertension, self-management including telemonitoring is more effective than usual care for reducing systolic blood pressure at 6 and 12 months. Evid Based Med 2011; 16:17–18. 21109677 10.1136/ebm1148

[24] Godwin M, Lam M, Birtwhistle R, Delva D, Seguin R, Casson I, MacDonald S A primary care pragmatic cluster randomized trial of the use of home blood pressure monitoring on blood pressure levels in hypertensive patients with above target blood pressure. Fam Pract 2010; 27:135–142. 20032170 10.1093/fampra/cmp094

[25] Green BB, Anderson ML, Cook AJ, Catz S, Fishman PA, McClure JB, Reid RJ e-Care for heart wellness: a feasibility trial to decrease blood pressure and cardiovascular risk. Am J Prev Med 2014; 46:368–377. 24650839 10.1016/j.amepre.2013.11.009 PMC3978093

[26] Green BB, Ralston JD, Fishman PA, Catz SL, Cook A, Carlson J, Tyll L, Carrell D, Thompson RS Electronic communications and home blood pressure monitoring (e-BP) study: design, delivery, and evaluation framework. Contemp Clin Trials 2008; 29:376–395. 17974502 10.1016/j.cct.2007.09.005 PMC2645352

[27] Halme L, Vesalainen R, Kaaja M, Kantola I; HOme MEasuRement of blood pressure study group Self-monitoring of blood pressure promotes achievement of blood pressure target in primary health care. Am J Hypertens 2005; 18:1415–1420. 16280273 10.1016/j.amjhyper.2005.05.017

[28] Hebert PL, Sisk JE, Tuzzio L, Casabianca JM, Pogue VA, Wang JJ, Chen Y, Cowles C, McLaughlin MA Nurse-led disease management for hypertension control in a diverse urban community: a randomized trial. J Gen Intern Med 2012; 27:630–639. 22143452 10.1007/s11606-011-1924-1 PMC3358388

[29] Kerry SM, Markus HS, Khong TK, Cloud GC, Tulloch J, Coster D, Ibison J, Oakeshott P Home blood pressure monitoring with nurse-led telephone support among patients with hypertension and a history of stroke: a community-based randomized controlled trial. CMAJ 2013; 185:23–31. 23128283 10.1503/cmaj.120832 PMC3537777

[30] Leiva A, Aguiló A, Fajó-Pascual M, Moreno L, Martín MC, Garcia EM, Duro RE, Serra F, Dagosto P, Iglesias-Iglesias AA, Company RM, Yañez A, Llobera J Efficacy of a brief multifactorial adherence-based intervention in reducing blood pressure: a randomized clinical trial. Patient Prefer Adherence 2014; 8:1683–1690. 25525344 10.2147/PPA.S66927 PMC4266385

[31] Magid DJ, Olson KL, Billups SJ, Wagner NM, Lyons EE, Kroner BA A pharmacist-led, American Heart Association Heart360 Web-enabled home blood pressure monitoring program. Circ Cardiovasc Qual Outcomes 2013; 6:157–163. 23463811 10.1161/CIRCOUTCOMES.112.968172

[32] Margolis KL, Asche SE, Bergdall AR, Dehmer SP, Groen SE, Kadrmas HM, Kerby TJ, Klotzle KJ, Maciosek MV, Michels RD, O’Connor PJ, Pritchard RA, Sekenski JL, Sperl-Hillen JM, Trower NK Effect of home blood pressure telemonitoring and pharmacist management on blood pressure control: a cluster randomized clinical trial. JAMA 2013; 310:46–56. 23821088 10.1001/jama.2013.6549 PMC4311883

[33] McKinstry B, Hanley J, Wild S, Pagliari C, Paterson M, Lewis S, Sheikh A, Krishan A, Stoddart A, Padfield P Telemonitoring based service redesign for the management of uncontrolled hypertension: multicentre randomised controlled trial. BMJ 2013; 346:f3030. 23709583 10.1136/bmj.f3030 PMC3663293

[34] McManus RJ, Mant J, Bray EP, Holder R, Jones MI, Greenfield S, Kaambwa B, Banting M, Bryan S, Little P, Williams B, Hobbs FD Telemonitoring and self-management in the control of hypertension (TASMINH2): a randomised controlled trial. Lancet 2010; 376:163–172. 20619448 10.1016/S0140-6736(10)60964-6

[35] McManus RJ, Mant J, Roalfe A, Oakes RA, Bryan S, Pattison HM, Hobbs FD Targets and self monitoring in hypertension: randomised controlled trial and cost effectiveness analysis. BMJ 2005; 331:493. 16115830 10.1136/bmj.38558.393669.E0 PMC1199029

[36] Parati G, Omboni S, Albini F, Piantoni L, Giuliano A, Revera M, Illyes M, Mancia G; TeleBPCare Study Group Home blood pressure telemonitoring improves hypertension control in general practice. The TeleBPCare study. J Hypertens 2009; 27:198–203. 19145785 10.1097/hjh.0b013e3283163caf

[37] Parati G, Omboni S, Compare A, Grossi E, Callus E, Venco A, Destro M, Villa G, Palatini P, Rosei EA, Scalvini S, Taddei S, Manfellotto D, Favale S, De Matteis C, Guglielmi M, Lonati L, Della Rosa F, Tosazzi E, Grandi AM, Maresca AM, Mongiardi C, Mare M, Ricci AR, Cagnoni F, Georgatos J, Besostri V, Ferrari V, Omodeo O, Dorigatti F, Bonso E, Guarnieri C, Muiesan L, Paini A, Stassaldi D, Cinelli A, Bernocchi P, Rocchi S, Magagna A, Ghiadoni L, Del Frate I, Boresi F, Guidi A, Re MA, Pellicciotti L, Florio A, Morani G, Di Lillo S, Ambrosio A, Casciello A, Quaglia M, Forleo C, Ardito MA, Gerunda S, Panunzio M; TELEBPMET Study Group Blood pressure control and treatment adherence in hypertensive patients with metabolic syndrome: protocol of a randomized controlled study based on home blood pressure telemonitoring vs. conventional management and assessment of psychological determinants of adherence (TELEBPMET Study). Trials 2013; 14:22. 23343138 10.1186/1745-6215-14-22 PMC3576326

[38] Stewart K, George J, Mc Namara KP, Jackson SL, Peterson GM, Bereznicki LR, Gee PR, Hughes JD, Bailey MJ, Hsueh YA, McDowell JM, Bortoletto DA, Lau R A multifaceted pharmacist intervention to improve antihypertensive adherence: a cluster-randomized, controlled trial (HAPPy trial). J Clin Pharm Ther 2014; 39:527–534. 24943987 10.1111/jcpt.12185

[39] Verberk WJ, Thien T, Kroon AA, Lenders JW, van Montfrans GA, Smit AJ, de Leeuw PW Prevalence and persistence of masked hypertension in treated hypertensive patients. Am J Hypertens 2007; 20:1258–1265. 18047914 10.1016/j.amjhyper.2007.08.002

[40] Wakefield BJ, Holman JE, Ray A, Scherubel M, Adams MR, Hillis SL, Rosenthal GE Effectiveness of home telehealth in comorbid diabetes and hypertension: a randomized, controlled trial. Telemed J E Health 2011; 17:254–261. 21476945 10.1089/tmj.2010.0176

[41] Yi SS, Tabaei BP, Angell SY, Rapin A, Buck MD, Pagano WG, Maselli FJ, Simmons A, Chamany S Self-blood pressure monitoring in an urban, ethnically diverse population: a randomized clinical trial utilizing the electronic health record. Circ Cardiovasc Qual Outcomes 2015; 8:138–145. 25737487 10.1161/CIRCOUTCOMES.114.000950 PMC4366280

[42] Ogedegbe G, Tobin JN, Fernandez S, Cassells A, Diaz-Gloster M, Khalida C, Pickering T, Schwartz JE Counseling African Americans to Control Hypertension: cluster-randomized clinical trial main effects. Circulation 2014; 129:2044–2051. 24657991 10.1161/CIRCULATIONAHA.113.006650 PMC4565195

[43] Bray EP, Holder R, Mant J, McManus RJ Does self-monitoring reduce blood pressure? Meta-analysis with meta-regression of randomized controlled trials. Ann Med 2010; 42:371–386. 20504241 10.3109/07853890.2010.489567

[44] Zimbudzi E, Lo C, Misso ML, Ranasinha S, Kerr PG, Teede HJ, Zoungas S Effectiveness of self-management support interventions for people with comorbid diabetes and chronic kidney disease: a systematic review and meta-analysis. Syst Rev 2018; 7:84. 29898785 10.1186/s13643-018-0748-z PMC6001117

[45] Bridgwood B, Lager KE, Mistri AK, Khunti K, Wilson AD, Modi P Interventions for improving modifiable risk factor control in the secondary prevention of stroke. Cochrane Database Syst Rev 2018; 5:CD009103. 10.1002/14651858.CD009103.pub3 PMC6494626 29734470

