# Supplementary material for: Self-monitoring of Blood Pressure in Patients With Hypertension-Related Multi-morbidity: Systematic Review and Individual Patient Data Meta-analysis
Source: Am J Hypertens. 2019 Nov 15;33(3):243–51. doi: 10.1093/ajh/hpz182 (PMC7162426; doi:10.1093/ajh/hpz182)
Supplement: hpz182_suppl_Supplementary_Appendix [file hpz182_suppl_supplementary_appendix.docx]

**Self-monitoring of blood pressure in patients with hypertension related multi-morbidity: Systematic review and individual patient data meta-analysis**

**Online supplemental material**

JP Sheppard*,* KL Tucker*,* WJ Davison, R Stevens*,* W Aekplakorn, HB Bosworth, A Bove, K Earle, M Godwin*,* BB Green*,* P Hebert, C Heneghan, N Hill*,* FDR Hobbs, I Kantola*,* SM Kerry*,* A Leiva, DJ Magid*,* J Mant, KL Margolis*,* B McKinstry*,* MA McLaughlin, K McNamara, S Omboni*,* O Ogedegbe*,* G Parati*,* J Varis*,* WJ Verberk*,* BJ Wakefield*,* RJ McManus

**Corresponding Author:** Katherine Tucker, [katherine.tucker@phc.ox.ac.uk](mailto:katherine.tucker@phc.ox.ac.uk)

**Contents**

1. **eFigure 1.** Example Search Strategy (Medline)
2. **eFigure 2**. Flow diagram of the update to the systematic search and selection of relevant studies.
3. **eTable 1.** Characteristics of included studies
4. **eTable 2.** Baseline characteristics of included studies (with outcomes at 6 and/or 12 months)
5. **eFigure 3.** Effect of self-monitoring on clinic systolic blood pressure at 12-month follow-up by intervention intensity
6. **eFigure 4.** Effect of self-monitoring on clinic diastolic blood pressure at 12-month follow-up by intervention intensity
7. **eFigure 5.** Effect of self-monitoring on clinic systolic blood pressure at 12-month follow-up by intervention intensity, including aggregate data from studies (n=5) not contributing individual patient data
8. **eFigure 6.** Funnelplot showing mean change in systolic blood pressure at 12 months
9. **eFigure 7.** Effect of self-monitoring on clinic blood pressure at 6-month follow-up by number of hypertension related co-morbidities
10. **eFigure 8.** Effect of self-monitoring on clinic diastolic blood pressure at 12-month follow-up by intervention intensity within specific morbidities
11. **eFigure 9.** Effect of self-monitoring on clinic systolic blood pressure at 6-month follow-up by intervention intensity within specific morbidities
12. **eFigure 10.** Effect of self-monitoring on clinic diastolic blood pressure at 6-month follow-up by intervention intensity within specific morbidities
13. **eFigure 11.** Effect of self-monitoring on likelihood of uncontrolled clinic blood pressure at 6-month follow-up by intervention intensity within specific morbidities

**eFigure 1.** Example Search Strategy (Medline)

Database: Ovid MEDLINE(R) Updated from original search to January 2018
Only trials published since 2000 eligible.

--------------------------------------------------------------------------------
1 blood pressure monitoring, ambulatory/
2 ((blood pressure or bp) adj3 (24h or 24hr? or 24-h or 24-hr? or 24 hour? or ambulatory or determin$ or measur$ or monitoring or monitor$ or self-measur$ or self-monitor$)).tw.
3 or/1-2
4 (home or self$).tw.
5 (telemedicine or tele-medicine or telemonitor$ or tele-monitor$).mp.

6 or/4-

7 randomized controlled trial.pt.
8 controlled clinical trial.pt.
9 randomized.ab.
10 placebo.ab.
11 drug therapy.fs.
12 randomly.ab.
13 trial.ab.
14 groups.ab.
15 or/7-14
16 animals/ not (humans/ and animals/)
17 15 not 16
18 3 and 6 and 17

**eFigure 2**. Flow diagram of the update to the systematic search and selection of relevant studies.

**Database search**

1377 unique studies identified

**Full text screening**

32 studies

120 duplicates

1225 studies not relevant on basis of titles and abstract screening

**Studies approached to participate**

3 studies

**36**

3636 studies

2 study authors did not respond or were not able to collaborate

**Studies included in analysis**

26 studies

25 studies included from previous search

**29** studies excluded:

**9** self-monitoring in control group

**5** not hypertensive

**4** abstract only

**3** trial already included

**2** no data

**2** not randomised trial

**1** not main study manuscript

**1** wrong comparator

**1** wrong intervention

**1** follow up not long enough

**Studies following up for 6-months**

22 studies

8,787 patients

**Studies following up for 12-months**

16 studies

7,360 patients

**Complete follow-up data**

7,601 patients

(87%)

**Complete follow-up data**

6,522 patients

(88.6%)

**eTable 1.** Characteristics of included studies

| Lead author/s  Country & year | Study | Self-monitoring | Co-interventions | Pre-defined level of intervention | Comparison | Home target  mmHg | Office target  mmHg | Baseline BP ±SD  mmHg | 6 m follow-up | 12 m follow-up | No. with multi-morbidity* | Morbidities recorded |
| --- | --- | --- | --- | --- | --- | --- | --- | --- | --- | --- | --- | --- |
| Halme/ Kantola[^18^](#_ENREF_18)  Finland, 2005 | HOMER | Daily for 1 week every 2 months | None | **Low intensity (level 1)** | Usual care | 135/80 | 140/85 | 157/93±18/8 | 170 | n/a | 85  (36.8%) | CHD  Stroke  Diabetes  Obesity |
| McManus[^19^](#_ENREF_19)  UK 2005 | TASMINH | Monthly in GP practice waiting room | None | **Low intensity (level 1)** | Usual Care | 140/85  140/80 DM | 140/85  140/80 DM | 156/88±15/7 | 413 | 401 | 238 (54.1%) | CHD  Stroke  Diabetes  Obesity |
| Bosworth[^20^](#_ENREF_20)  US, 2007 | HINTS | 3 days per week | Behavioural intervention  Or meds management  Or both | **High intensity (Level 3)**  **High intensity (level 4)** | Usual Care | 135/85  135/80 DM | 140/90  135/80 DM | 129/77±18/13  130/78±19/14  128/77±19/13 | 535 | 523 | 389 (65.8%) | CHD  Stroke  Diabetes  Obesity |
| Verberk[^21^](#_ENREF_21)  Netherlands, 2007 | HOMERUS | 1 week per month then 1 week every 2 months | None | **Low intensity (level 1)** | Usual care with monthly clinic visits then 2 monthly | 140/90 | 140/90 | 164/96±17/10 | n/a | 434 | 153 (29.6%) | CHD  Stroke  CKD  Obesity |
| Green[^22^](#_ENREF_22)  US, 2008 | eBP | 2 days per week | Website and email  +/- web based pharmacist management | **Low intensity (level 2)**  **High intensity (level 4)** | Usual care | 135/85 | 140/90 | 151/89±12/9 151/89±12/9 | n/a | 730 | 458  (58.9%) | CHD  Stroke  Diabetes  CKD  Obesity |
| Bosworth[^23^](#_ENREF_23)  US, 2009 | TCYB | 3 days per week | None  Or behavioural interventions, education and support | **Low intensity (level 1)**  **High intensity (level 4)** | Usual care | 135/85  130/80 DM | 140/90  130/80 DM | 125/71±16/10  125/71±18/11 | 383 | 350 | 187 (41.9%) | CHD  Stroke  Diabetes  CKD  Obesity |
| Parati & Omboni[^24^](#_ENREF_24)  Italy, 2009 | TeleBPcare | 3 days per week | Telemonitoring | **Low intensity (level 2)** | Usual care | 135/85 | 140/90 | 146/88±12/8 | 298 | n/a | 59  (19.8%) | CHD  Stroke  Diabetes  CKD  Obesity |
| Godwin[^25^](#_ENREF_25)  Canada, 2010 |  | At least weekly | None | **Low intensity (level 1)** | Usual Care | 135/85 | 140/90 | 144/81±18/11 | 458 | 458 | 251 (54.8%) | Diabetes  Obesity |
| Earle [^26^](#_ENREF_26)  UK, 2010 |  | weekly | Blood glucose testing, text/App system with feedback from clinicians | **High intensity (level 4)** | Usual Care | 140/90 | 140/90 | 131/77±17/10 | 126 | n/a | 127 (100%) | Diabetes  CKD |
| McManus[^27^](#_ENREF_27)  UK, 2010 | TASMINH2 | Daily for the first week of each month | Telemonitoring and self- titration | **High intensity (level 3)** | Usual care | 130/85  130/75 DM | 140/90  140/80 DM | 152/85±12/9 | 480 | 480 | 290 (55.0%) | CHD  Stroke  Diabetes  CKD  Obesity |
| Hebert[^28^](#_ENREF_28)  US, 2011 |  | Variable | None  Or nurse support | **Low intensity (level 1)**  **High intensity (level 4)** | Usual care |  | 140/90  130/80DM | 153/86 16/13  153/86 18/13 | n/a | n/a ** | 343 (82.5%) | Diabetes  CKD  Obesity |
| Wakefield[^29^](#_ENREF_29)  US, 2011 |  | Daily | Low intensity management algorithm  High intensity algorithm | **Low intensity (level 2)**  **High intensity (level 3)** | Usual Care | 135/85  130/80 DM | 135/85  130/80 DM | 135/72±18/11  136/74±19/11 | 268 | 261*** | 300 (100%) | Diabetes  Obesity |
| Bove^[30](#_ENREF_30)^  US, 2013 | HTN | 2 days per week | Telemonitoring | **Low intensity (level 2)** | Usual care |  | 140/90 | 155/88±15/11 | 202 | n/a | 170 (72.3%) | CHD  Stroke  Diabetes  Obesity |
| Kerry[^15^](#_ENREF_15)  UK, 2013 |  | Daily in week 1, then 1 day per week | Nurse led telephone support | **Low intensity (level 2)** | Usual care | 130/80 | 140/85 | 138/74±21/12 | 352 | 334 | 381 (100%) | CHD  Stroke  Diabetes  Obesity |
| Magid^[31](#_ENREF_31)^  US, 2013 |  | 3 days per week | Patient education and BP reporting or patient education, BP reporting and pharmacist management | **High intensity (level 4)** | Usual Care | 135/85  125/75 DM/CKD | 140/90  130/80DM/ CKD | 147/15±89/10 | 326 | n/a | 159 (48.8%) | Diabetes  CKD  Obesity |
| Margolis[^32^](#_ENREF_32)  US, 2013 | Hyperlink | 3 days per week | Telemonitoring and pharmacist management | **High intensity (level 4)** | Usual Care | 135/85  125/75 DM/CKD | 140/90  130/80 DM/CKD | 148/85±13/12 | 403 | 388 | 311 (69.1%) | CHD  Stroke  Diabetes  CKD  Obesity |
| McKinstry^[33](#_ENREF_33)^  UK, 2013 | HITS | Daily in week 1, then at least 1 day per week thereafter | Optional automated telemonitoring | **Low intensity (level 2)** | Usual Care | 135/85 | 140/90 | 153/91±15/11 | 374 | n/a | 168 (41.9%) | Stroke  Diabetes  CKD  Obesity |
| Parati[^34^](#_ENREF_34)  Italy, 2013 | TeleBPMET | 3 days per week | Telemonitoring | **Low intensity (level 2)** | Usual Care | 135/85 | 140/90 | 147/90±12/8 | 181 | 179 | 116 (63.7%) | CHD  Stroke  Diabetes  CKD  Obesity |
| Green[^35^](#_ENREF_35)  US, 2014 | eCare | At least 1 day per week for 2 months, 1 day per fortnight for 2 months, then monthly | Dietician with BP plan and visits (weekly for 2 months, fortnightly for 2 months then monthly) | **High intensity (level 4)** | Usual care | 135/85 | 140/90 | 150/92±12/9 | 90 | n/a | 72  (71.3%) | CHD  Stroke  Diabetes  CKD  Obesity |
| Leiva [^36^](#_ENREF_36)  Spain, 2014 | Adherencia | Weekly, with morning and afternoon readings. | Motivational interview, pillbox reminder, Family support, BP and medication reminder form and pharmacist review | **High intensity (level 3)** | Usual Care | 135/85 | 140/90 and 130/80 for DM or CKD | 156/84±15/11 | n/a | 214 | 164 (76.6%) | CHD  Stroke  Diabetes  CKD  Obesity |
| McManus[^7^](#_ENREF_7)  UK 2014 | TASMIN-SR | Daily for the first week of each month | Self-management | **High intensity (level 3)** | Usual Care | 120/75 | 130/80 ST | 144/80±13/10 | 439 | 450 | 450 (100%) | CHD  Stroke  Diabetes  CKD  Obesity |
| Ogedegbe[^37^](#_ENREF_37)  US, 2014 | CAATCH | 3 days per week | Education, lifestyle and behavioural support | **High intensity (level 3)** | Usual Care |  |  | 151/91±17/10 | 610 | 691 | 656 (65.8%) | CHD  Stroke  Diabetes  CKD  Obesity |
| Stewart [^38^](#_ENREF_38)  Australia, 2014 | HAPPy | Several readings per week | Pharmacist management with motivational interviewing, medication review, education and optional refill reminders | **High intensity (level 4)** | Usual Care | 140/90 and 130/80 for DM and CKD | 140/90 and 130/80 for DM and CKD | 141/84±20/11 | 348 | n/a | 208 (53.6%) | CHD  Stroke  Diabetes  CKD  Obesity |
| Yi [^39^](#_ENREF_39)  US, 2015 |  | As prescribed by their doctor | Educational material on hypertension | **Low intensity (level 1)** | Usual Care | 140/90 or 130/80 DM or CKD | 140/90 or 130/80 DM or CKD | 152/83±16/11 | 529 | n/a | 723 (87.3%) | CHD  Stroke  Diabetes  CKD  Obesity |
| Parati  Italy | AUPRES | 3 days per week |  | **Low intensity (level 1)** | Usual Care | 135/85 | 140/90 | 154/95±15/8 | 407 | 407 | 55  (13.5%) | CHD  Stroke  Diabetes  CKD  Obesity |
| Aekplakorn^16^  Thailand, 2016 |  | Twice daily for 6 months | Clinic visits to discuss BP status and advice on medication and healthy  lifestyle. | **High intensity (level 4)** | Usual Care | 140/90 | 140/90 | 148/83 | 209 | 222 | 69  (30.8%) | CHD  Stroke  Diabetes  Obesity |

*Multi-morbidity defined as the presence of two or more morbidities (this study focused on cardiovascular multi-morbidity: Hypertension, coronary heart disease, stroke/transient ischemic attack, diabetes, chronic kidney disease and obesity).

**Study collected outcome data at 18 months.

***Participants self-monitored for 6 months, follow up data collected at 12 months.

**eTable 2.** Baseline characteristics of included studies (with outcomes at 6 and/or 12 months)

| Characteristic | Total population (hypertensives)* | | CHD | | Stroke | | Diabetes | | CKD | | Obesity | |
| --- | --- | --- | --- | --- | --- | --- | --- | --- | --- | --- | --- | --- |
|  | Mean/  number | SD/IQR/% | Mean/  number | SD/IQR/% | Mean/  number | SD/IQR/% | Mean/  number | SD/IQR/% | Mean/  number | SD/IQR/% | Mean/  number | SD/IQR/% |
| Total population | 10,713 |  | 827 |  | 789 |  | 2964 |  | 650 |  | 4,368 |  |
| Age (years) | 62.7 | 11.7 | 67.7 | 10.9 | 68.6 | 12.2 | 63.0 | 11.0 | 64.9 | 11.5 | 59.8 | 11.0 |
| Sex (% Male) | 5,891 | 55.0% | 459 | 55.5% | 440 | 55.8% | 1,789 | 60.4% | 361 | 55.5% | 2,363 | 54.1% |
| BMI (kg/m2) | 30.6 | 6.4 | 30.5 | 6.5 | 28.5 | 5.9 | 32.7 | 7.1 | 31.3 | 6.6 | 35.8 | 5.5 |
| Smoking (%, yes) | 1,342 | 15.9% | 56 | 14.6% | 102 | 16.3% | 341 | 18.9% | 58 | 10.5% | 507 | 14.5% |
| Alcohol (median, units per week) | 2.0 | 0.0, 9.5 | 1.5 | 0.0, 9.0 | 0.0 | 0.0, 6.0 | 1.0 | 0.0, 7.8 | 2.0 | 0.0, 8.5 | 2.0 | 0.0 |
| Clinic Systolic BP (mmHg) | 147.6 | 18.3 | 147.9 | 18.8 | 145.3 | 20.9 | 144.8 | 19.7 | 147.6 | 17.8 | 147.8 | 17.6 |
| Clinic Diastolic BP (mmHg) | 85.3 | 12.2 | 80.8 | 11.6 | 79.2 | 12.8 | 80.8 | 12.6 | 83.0 | 11.5 | 85.7 | 11.9 |
| BP meds at baseline | 1.8 | 1.2 | 2.0 | 1.0 | 1.8 | 1.1 | 2.2 | 1.3 | 1.9 | 1.0 | 1.9 | 1.2 |

*All included participants had to have at least one morbidity (hypertension) to be included in each original trial

CHD=coronary heart disease; CKD=chronic kidney disease; SD=standard deviation; IQR=inter quartile range; BMI=body mass index; BP=blood pressure

**eFigure 3.** Effect of self-monitoring on clinic systolic blood pressure at 12-month follow-up by intervention intensity

**
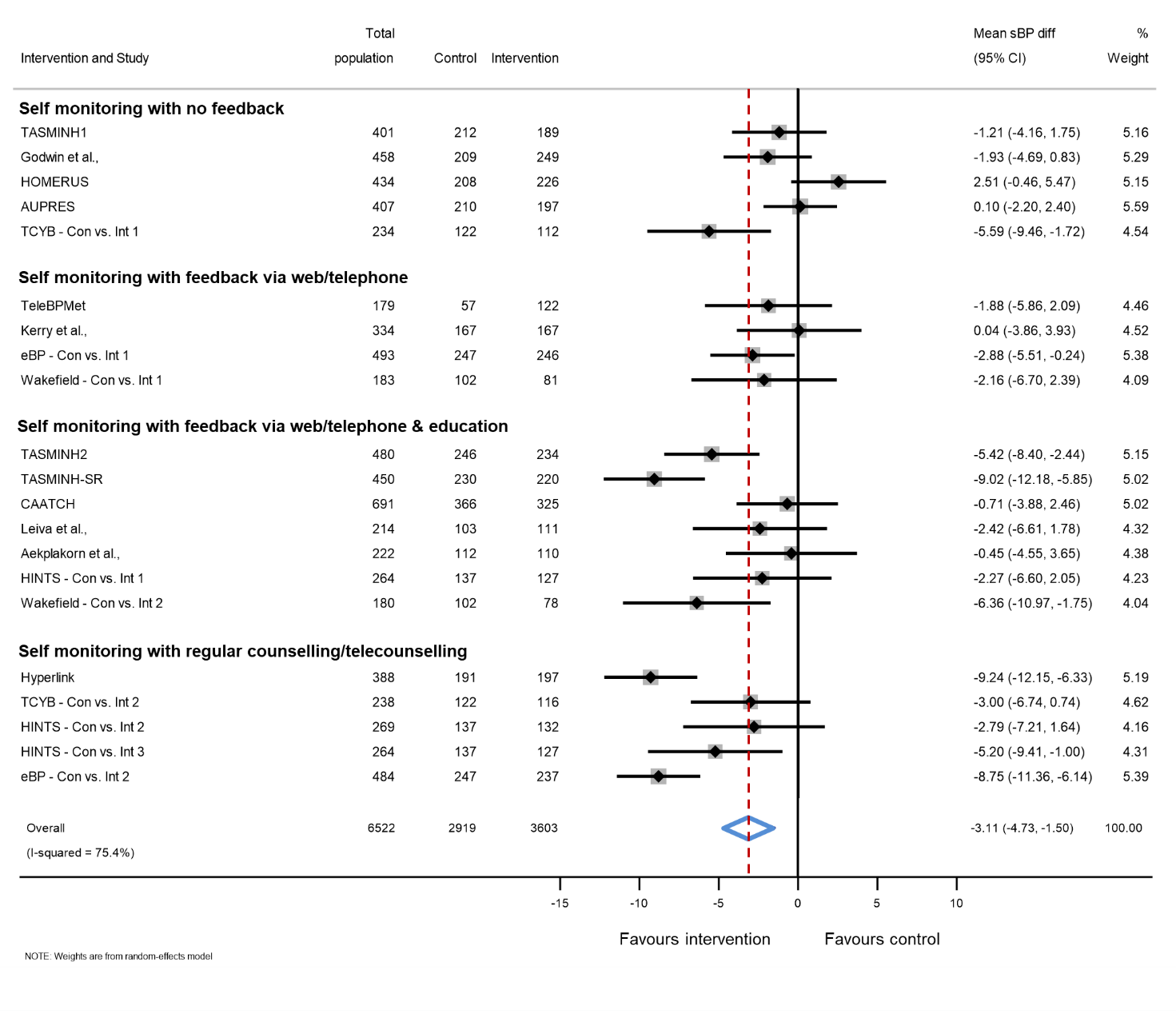
**

Analyses adjusted for age, sex, baseline systolic blood pressure and diabetes. sBP=systolic blood pressure; CI=confidence intervals

**eFigure 4.** Effect of self-monitoring on clinic diastolic blood pressure at 12-month follow-up by intervention intensity


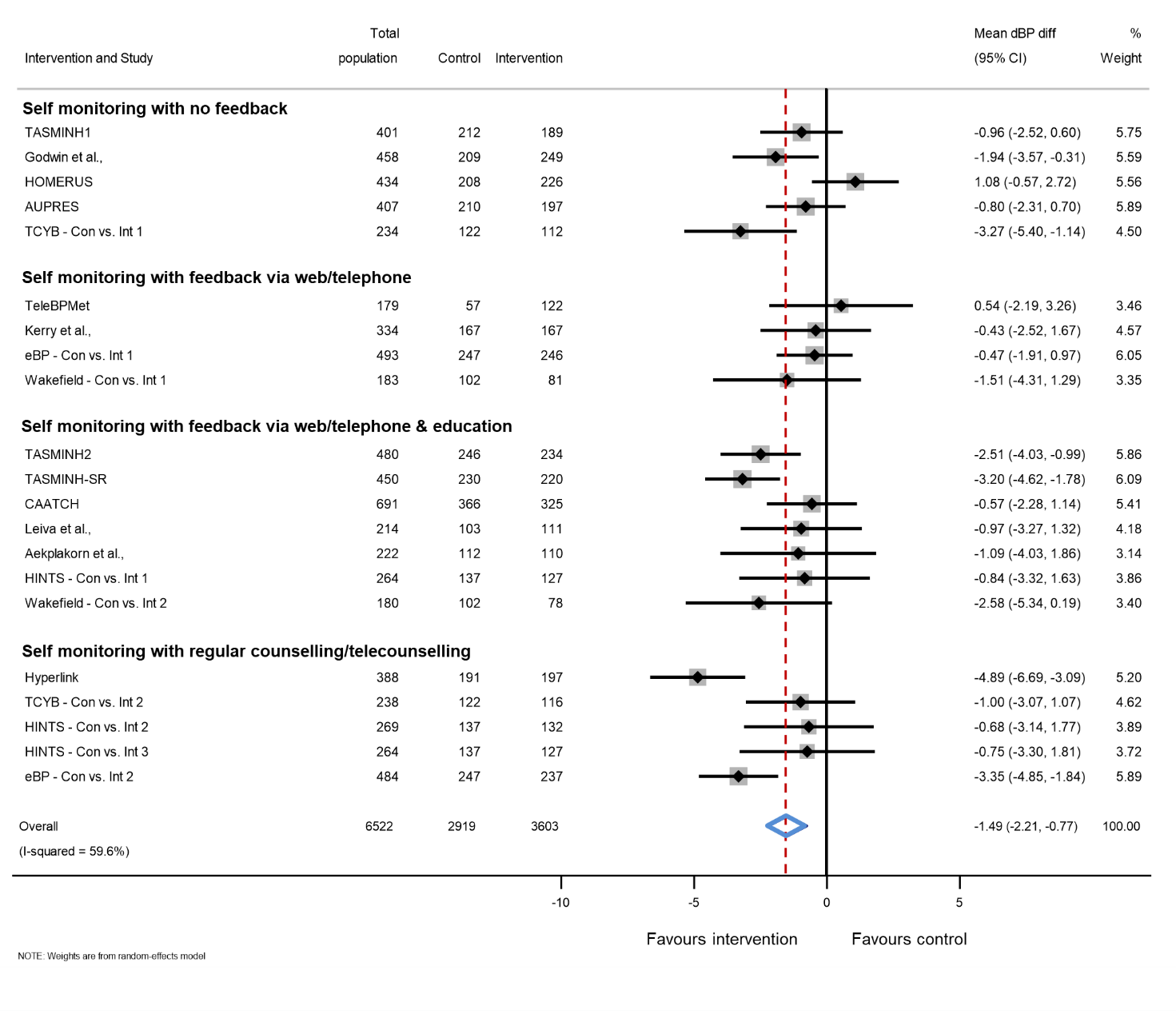


Analyses adjusted for age, sex, baseline diastolic blood pressure and diabetes. dBP=diastolic blood pressure; CI=confidence intervals

**eFigure 5.** Effect of self-monitoring on clinic systolic blood pressure at 12-month follow-up by intervention intensity, including aggregate data from studies (n=5) not contributing individual patient data


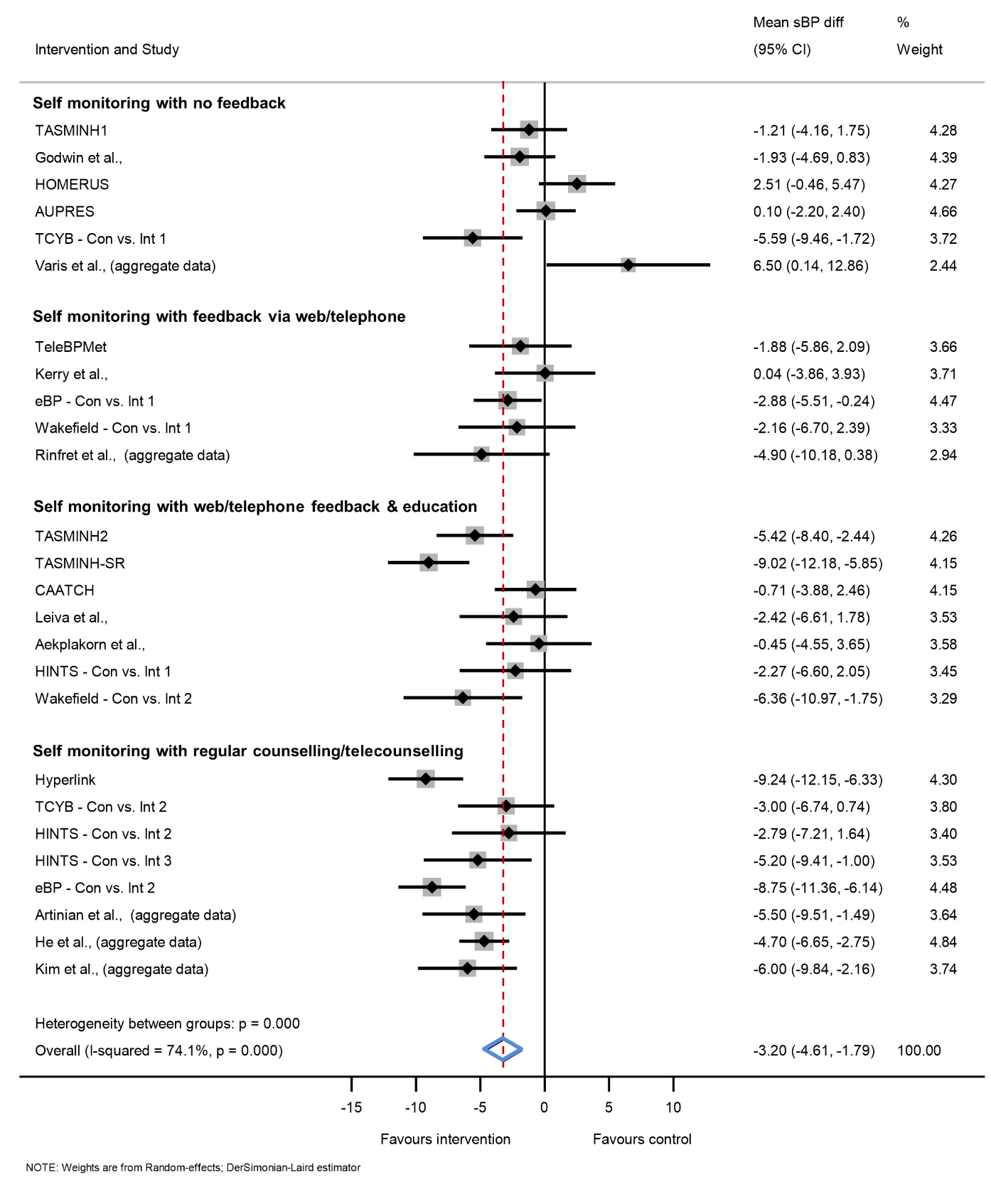


One eligible study which did not provide individual patient data (Qi *et al., Biomedical Research,* 2017;28: 2898-2902) could not be included in this figure due to a lack of data on the variance (SE or SD) around the mean systolic blood pressure change at 12 month follow-up. Analyses adjusted for age, sex, baseline systolic blood pressure and diabetes. sBP=systolic blood pressure; CI=confidence intervals

**eFigure 6.** Funnelplot showing mean change in systolic blood pressure at 12 months


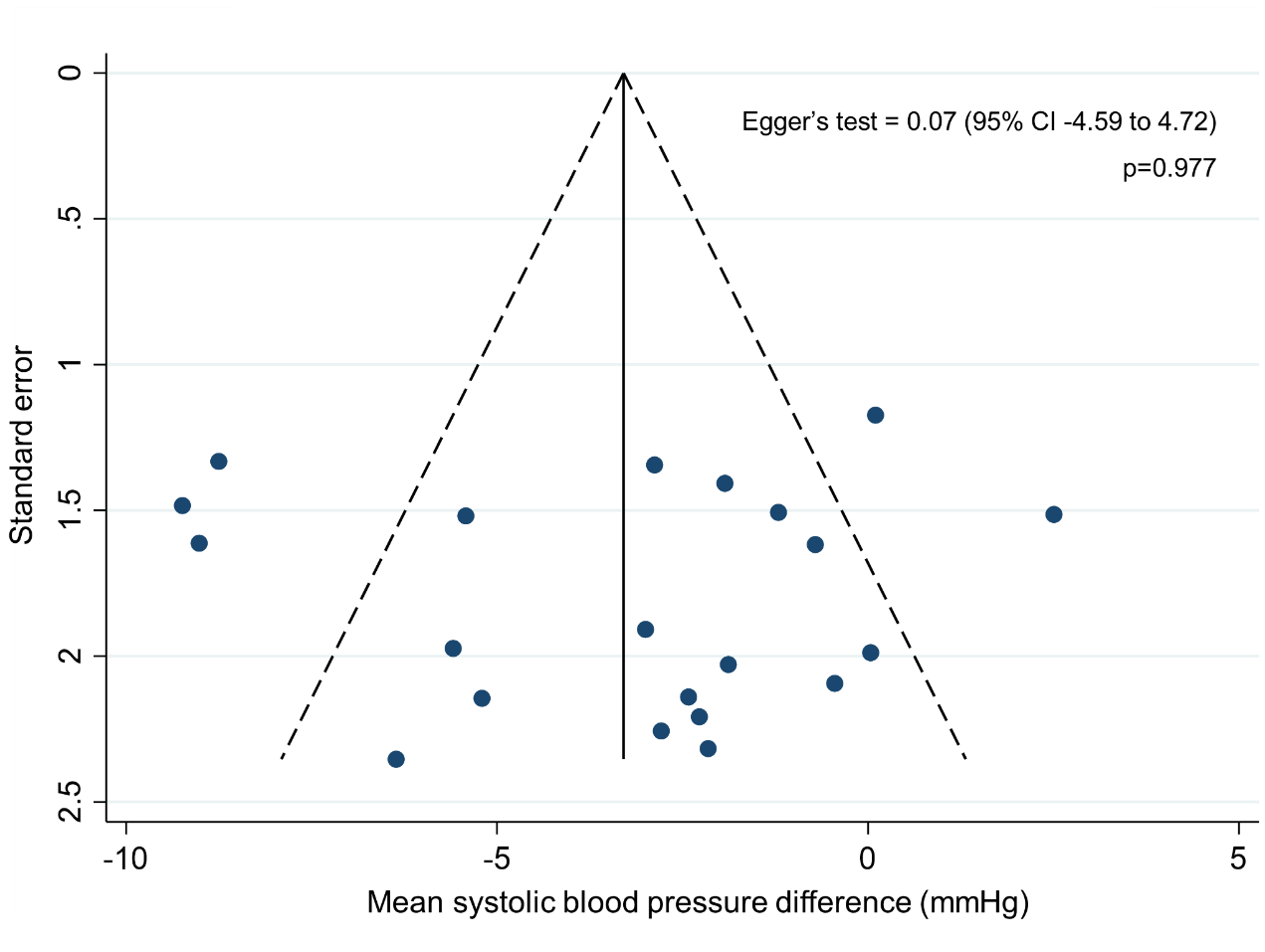


The standard error is plotted in against the mean change in systolic blood pressure at 12 months. An Egger’s test of zero (p=1.00) would indicate no influence of publication bias

**eFigure 7.** Effect of self-monitoring on blood pressure at 6-month follow-up by number of hypertension related co-morbidities (22 studies)

**
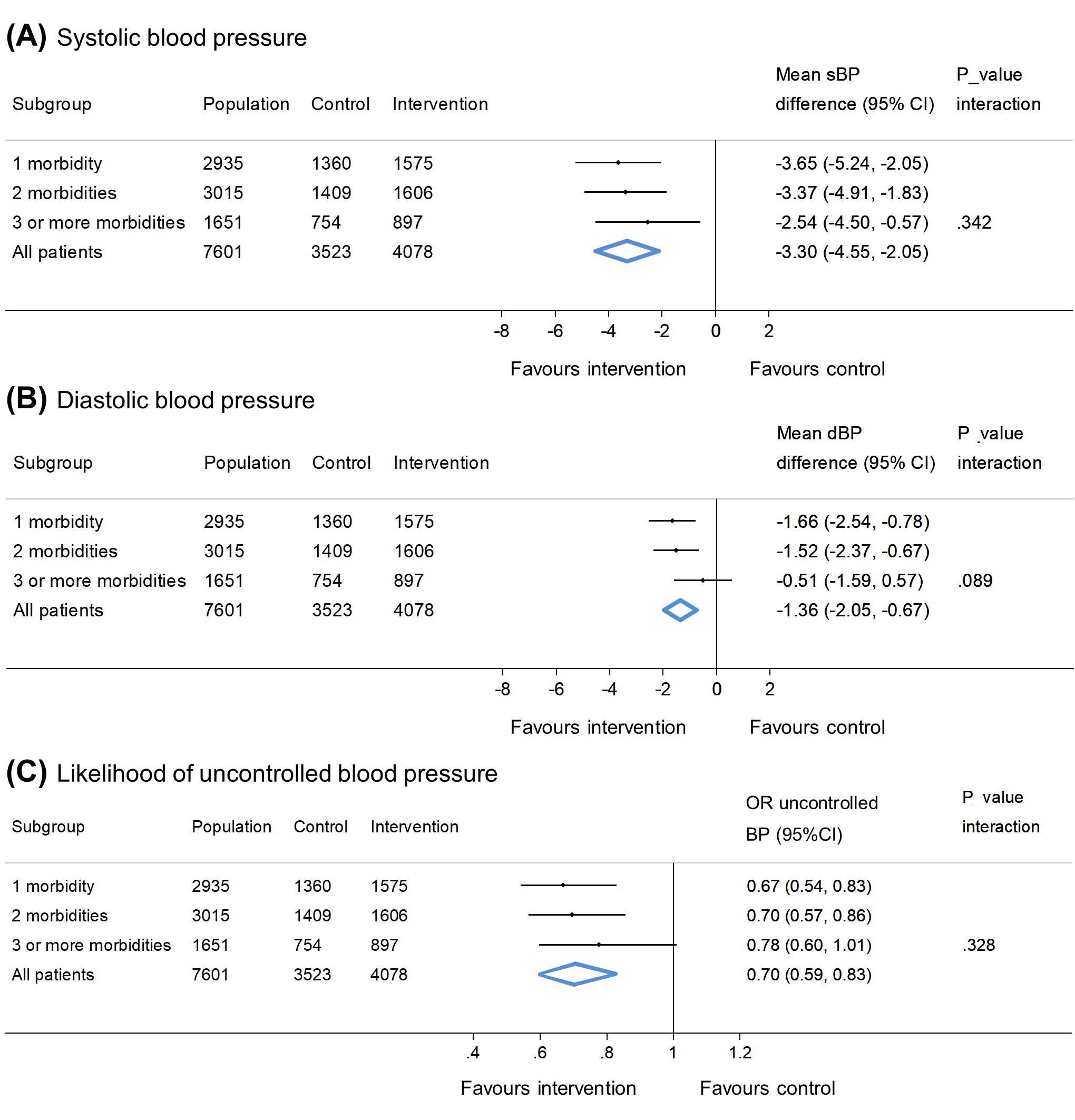
**

Blood pressure difference given in mm Hg. Analyses adjusted for age, sex, baseline blood pressure and level of intervention, with study level random effects for intervention and usual care

sBP=systolic blood pressure; dBP=diastolic blood pressure; CI=confidence intervals; OR=odds ratio

**eFigure 8.** Effect of self-monitoring on clinic diastolic blood pressure at 12-month follow-up by intervention intensity within specific morbidities


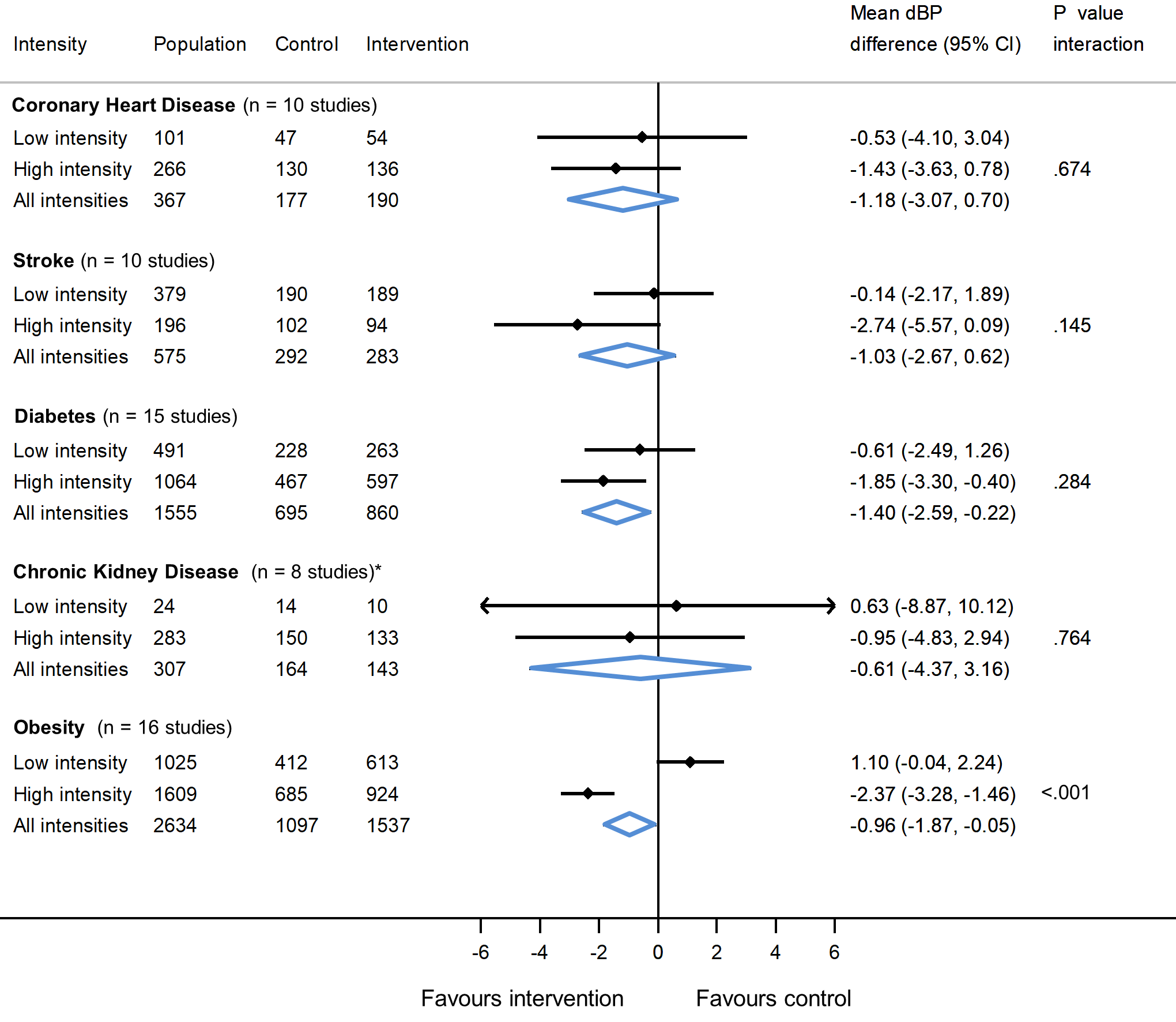


*Two studies only provided one patient each to the model. Blood pressure difference given in mm Hg. Analyses adjusted for age, sex and baseline blood pressure with study level random effects for intervention and usual care. dBP=diastolic blood pressure; CI=confidence intervals; CHD=coronary heart disease; CKD=chronic kidney disease

**eFigure 9.** Effect of self-monitoring on systolic blood pressure at 6-month follow-up by intervention intensity within specific morbidities


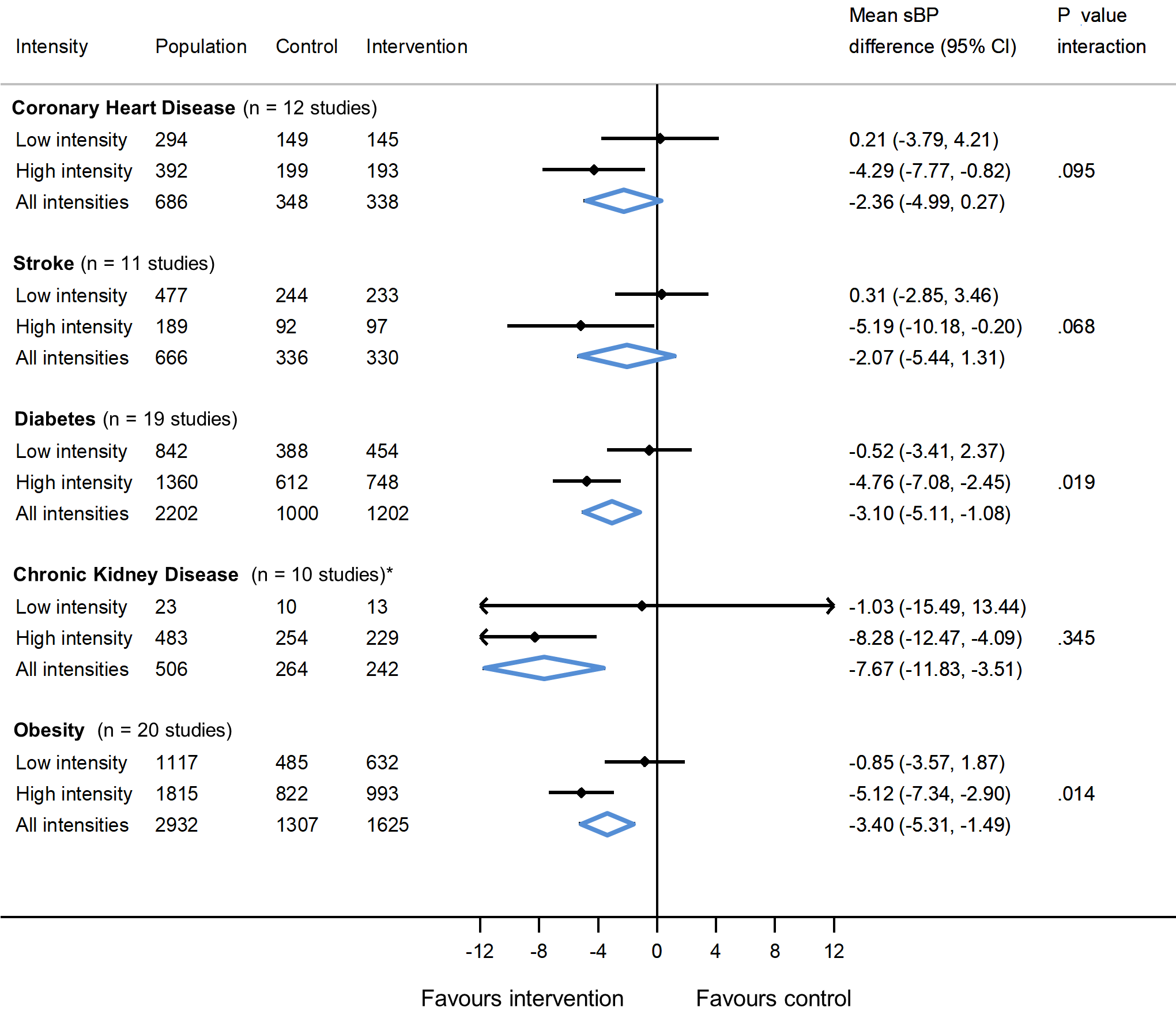


*Two studies only provided one patient each to the model. Blood pressure difference given in mm Hg. Analyses adjusted for age, sex and baseline blood pressure with study level random effects for intervention and usual care. sBP=systolic blood pressure; CI=confidence intervals; CHD=coronary heart disease; CKD=chronic kidney disease

**eFigure 10.** Effect of self-monitoring on diastolic blood pressure at 6-month follow-up by intervention intensity within specific morbidities


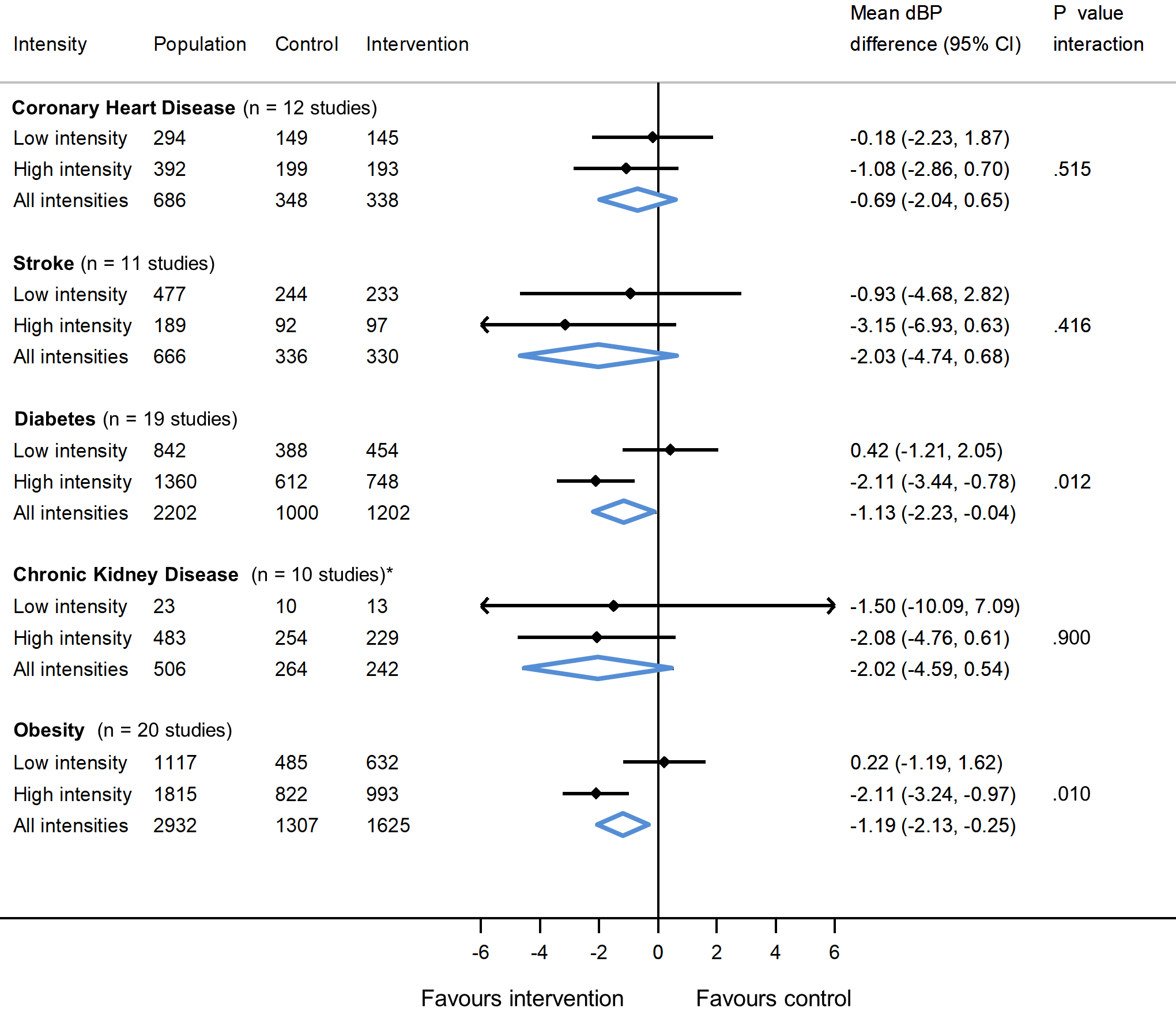


*Two studies only provided one patient each to the model. Blood pressure difference given in mm Hg. Analyses adjusted for age, sex and baseline blood pressure with study level random effects for intervention and usual care. dBP=diastolic blood pressure; CI=confidence intervals; CHD=coronary heart disease; CKD=chronic kidney disease

**eFigure 11.** Effect of self-monitoring on likelihood of uncontrolled blood pressure at 6-month follow-up by intervention intensity within specific morbidities


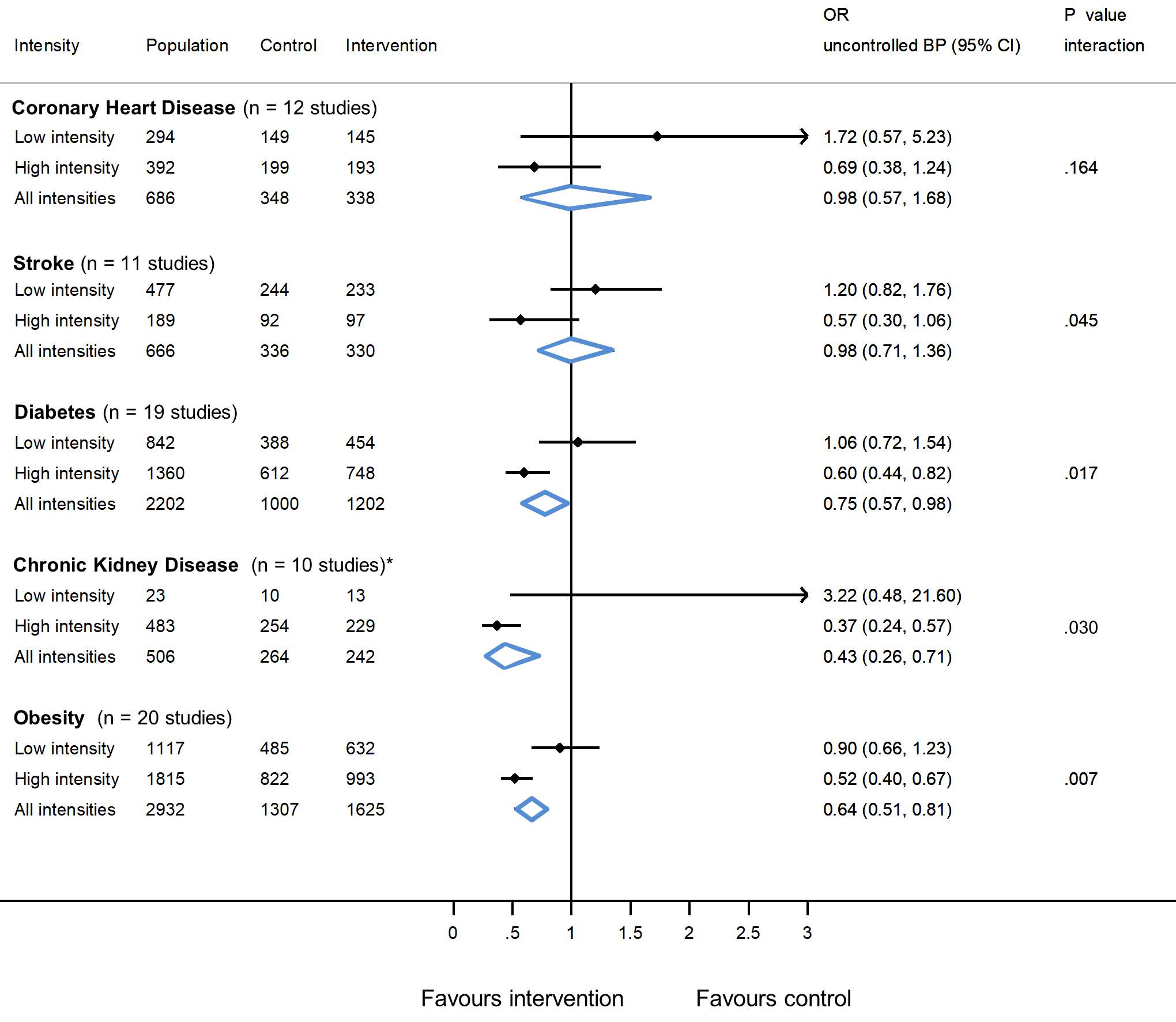


*Two studies only provided one patient each to the model. Analyses adjusted for age, sex and baseline blood pressure with study level random effects for intervention and usual care. OR=odds ratio; CI=confidence intervals; CHD=coronary heart disease; CKD=chronic kidney disease
